# Review of Laser Powder Bed Fusion’s Microstructure and Mechanical Characteristics for Al-Ce Alloys

**DOI:** 10.3390/ma17205085

**Published:** 2024-10-18

**Authors:** Yuanfan Liu, Yang Li, Mingliang Wang, Zhe Chen

**Affiliations:** 1 SJTU-Paris Tech Elite Institute of Technology (SPEIT), Shanghai 200240, China; wat3ble@sjtu.edu.cn; 2 State Key Laboratory of Metal Matrix Composites, Shanghai Jiao Tong University, Shanghai 200240, China; liyang772@sjtu.edu.cn; 3 School of Materials Science & Engineering, Shanghai Jiao Tong University, Shanghai 200240, China; 4 Institute of Alumics Materials, Shanghai Jiao Tong University (Anhui), Huaibei 235000, China; 5 Anhui Province Industrial Generic Technology Research Center for Alumics Materials, Huaibei Normal University, Huaibei 235000, China

**Keywords:** laser powder bed fusion, Al-Ce alloys, high temperature, mechanical properties

## Abstract

As a new alloy manufacturing method that can break through the limitations of molds to manufacture fine parts, laser powder bed fusion has recently become a common process for producing aluminum alloys. In the fields of aerospace or automotive, aluminum alloys with both good printability and good mechanical performance in high-temperature conditions are greatly demanded, and the Al-Ce alloy is one of the alloys with significant potential. Therefore, systematic research on the additive manufacturing of Al-Ce alloys is still being explored. Herein, we review the recent progress and current status of laser powder bed fusion-produced Al-Ce alloys, giving our opinions on the development of this alloy system. Element composition, alloy powders, laser powder bed fusion parameters, microstructures, and mechanical properties at room temperature and high temperatures are summarized. The choice of alloying strategies is crucial for a specific mechanical improvement of the Al-Ce alloys. Finally, the details of the Al-Ce alloys manufactured via laser powder bed fusion are presented.

## 1. Introduction

Additive manufacturing (AM), commonly referred to as 3D printing, encompasses a range of technologies that construct three-dimensional objects through a process of layer-by-layer fabrication, guided by digital models. Compared to traditional casting, AM can break through the limitations of mold and size, directly produce parts with extremely complex shapes [1,2], and enable rapid prototyping, thereby shortening the product development cycle [3]. Featuring a higher cooling rate, AM can produce parts with much more refined microstructures, which is promising for manufacturing parts with excellent mechanical performance [4,5,6]. In this way, AM has revolutionized the production of complex and lightweight components for various industries, offering enhanced design flexibility and cost-effective manufacturing [7,8,9]. Aluminum alloys are extensively utilized across various industrial sectors, including aerospace [10], automotive [11], and military applications [12]. Recently, a variety of AM technologies have been employed in the fabrication of aluminum alloys. These include laser additive manufacturing [13,14], wire arc additive manufacturing [15], friction stir welding additive manufacturing [16], ultrasonic additive manufacturing [17], and friction rolling additive manufacturing [18]. Among the various additive manufacturing techniques, laser powder bed fusion (LPBF) has garnered significant interest due to its ability to produce high-performance metallic components [19,20,21]. The LPBF process entails the continuous melting and solidification of the powder layer, as well as the underlying alloy, through the application of a rapidly moving laser. This method offers several benefits, including a high cooling rate, a significant thermal gradient, and the occurrence of repeated thermal cycles [2,22,23,24]. The LPBF technique has proven effective in the production of various metallic materials, particularly aluminum alloys. For instance, Uzan et al. [25] conducted an investigation into the mechanical properties of the AlSi10Mg alloy produced via LPBF. The findings indicated that the alloy exhibited a yield strength of 204 MPa, an ultimate tensile strength of 358 MPa, and an elongation of 7.2% at room temperature (RT). In another example, Shi et al. [26] fabricated an as-LPBFed 1 wt.%TiB_2_/AlSi10Mg composite. The fatigue limit of this composite reached 201 MPa, which was superior to other AlSi10Mg materials.

Despite the extensive utilization of aluminum alloys across various industrial applications, it is noteworthy that the majority of these alloys are primarily designed for use under RT conditions. Only a limited number of aluminum alloys possess the ability to withstand high-temperature (HT) environments. Although Al-Si alloys usually exhibit high strength at RT, their strength should decrease rapidly as the temperature increases. Roy et al. [27] investigated the thermophysical properties of five cast Al alloys. Their findings indicated that the prolonged heat treatment caused a drastic drop in the hardness of all the alloys, which was attributed to the significant coarsening of the strengthening precipitates. Sc and Zr have been employed as alloying constituents to maintain HT strength because they are capable of forming L1_2_-type Al_3_Sc and Al_3_Zr phases. These phases demonstrate slow coarsening kinetics and contribute to precipitation strengthening at HT [28,29].

Recently, Al-Ce-based alloys have garnered increasing interest due to their remarkable stability under HT conditions and their ability to maintain strength following exposure to such environments [30,31,32]. As the study of the Al-Ce alloys continues to progress, the research has been highly focused on their mechanical properties [33]. The benefits of Ce in Al-Ce-based alloys are multifaceted, including, but not limited to, the following: (1) Al-Ce alloys exhibit thermal stability owing to the minimal solubility of cerium (approximately 0.01 wt.% at RT) and its slow diffusivity within the aluminum matrix [34,35]; (2) the incorporation of cerium enhances the processability of aluminum alloys [36]; (3) cerium is the most abundant rare earth (RE) element, and its cost is significantly lower than that of other RE elements, often being regarded as a waste by-product [37]. Therefore, the study of the Al-Ce alloys is necessary when it comes to sustainable development. It is posited that Al-Ce-based alloys may prove advantageous for applications requiring lightweight materials and HT performance. Meanwhile, the Al–Ce binary system is basically outstanding in the strength aspect, although it is needed to develop Al-Ce-X ternary systems or other Al-Ce-based alloys, including Al-Ce-Mg [38,39,40], Al-Ce-Ni [41], Al-Ce-Cu [42], and Al-Ce-Si [43] alloys.

In this review, we investigate more than 100 published studies concerning Al-Ce-based alloys that have been produced using the LPBF technique, and we present a comprehensive summary of the elemental composition, alloy powders, LPBF strategies, microstructure, phase composition, and mechanical properties under both RT and HT conditions. It is our aspiration that this review will serve as a valuable resource for the continued advancement of LPBFed Al-Ce-based alloys.

## 2. Element Composition

**Figure 1:**
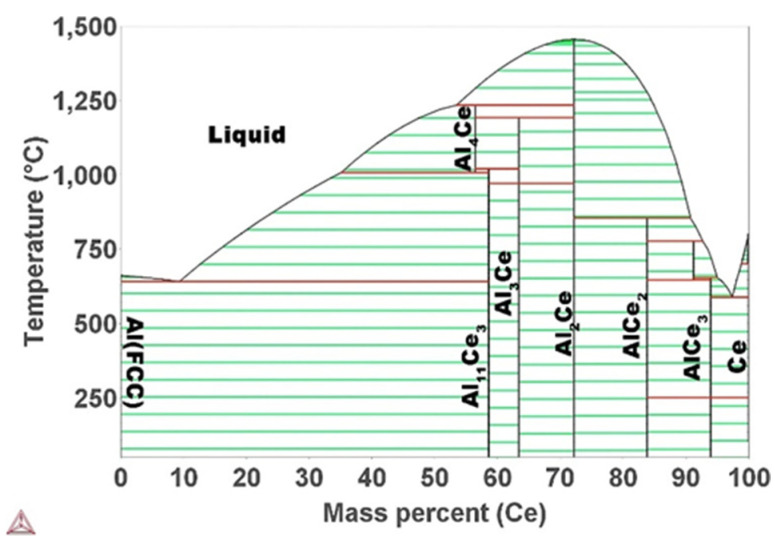
Al-Ce phase diagram computed utilizing the Calphad method [44].

In the context of developing new aluminum alloys for AM, Ce is infrequently examined, despite the benefits previously outlined. In 2016, Sims et al. [43] conducted the preliminary investigation on the conventional cast Al-Ce alloy to assess its potential for the advancement of a new series of aluminum alloys. The findings of this study indicate that the Al-Ce binary system demonstrates a eutectic reaction characterized by a relatively narrow solidification range. For example, Plotkowski et al. [44] employed the Calphad method to compute the phase diagram for Al-Ce system (Figure 1), from which the eutectic point was estimated to be Al-10 wt.% Ce with a melting point of 640 °C. Experimental investigations have yet to reach a consensus regarding the precise eutectic point of the Al-Ce binary system. Nevertheless, the weight percentage of Ce is considered, to date, to be between 10 and 12 wt.% [33,44,45]. The eutectic microstructure of the Al-Ce system usually consists of the lamellar α-Al and Al_11_Ce_3_ intermetallic compound [46,47]. Both phase composition and microstructure morphology can have a significant impact on the mechanical properties. It is then important to study the phase composition of the Al-Ce alloy. Even though the Al-Ce binary diagram is usually useful for cast alloys, it can no longer guide the manufacturing of LPBFed Al-Ce alloys. This is because the rapid solidification during AM can lead to severe distortion of the thermodynamic phase diagram [48].

**Figure 2:**
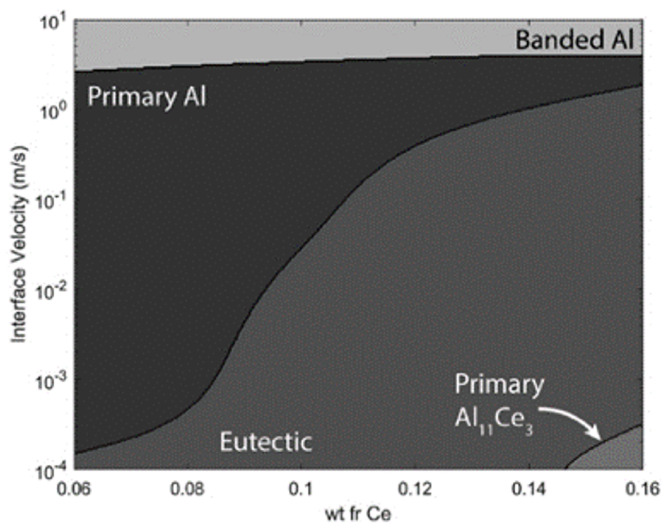
Al-Ce Microstructure selection map for composition/velocity with the thermal gradient of 10^6^ K/m for mass diffusivities of 10^−9^ m^2^/s [49].

In the following work, Plotkowski et al. [49] have conducted calculations and developed a microstructure selection map for additively manufactured Al-Ce alloys through laser melting experiments and assessments of analytical models. These models contained the dendritic growth theory by Kurz, Giovanola, and Trivedi [50] and the eutectic growth theory by Kurz and Trivedi [51] developed for the rapid solidification (Figure 2). There are differences in the microstructure features shown in the microstructure selection maps. For a certain nominal composition of Ce, the solidification process in the melt pools can be described as the laser goes from the edge to the center, which means that the increasing interface velocity can be represented by an arrow straight up in the selection maps.

In such cases [50], the selection of microstructures varies from eutectic to primary Al. Over the eutectic point of the Al-10 wt.% Ce alloy, a wide range of compositions are fairly stable at the lower interface growth velocities. At elevated velocities, a primary dendritic/cellular Al structure exhibits stability. Notably, in compositions that are abundant in cerium, the formation of primary intermetallic compounds is only feasible at significantly reduced velocities. This finding aligns with the observations made by Sims et al. [52] regarding the low interface growth velocities typically observed in casting processes. This research also showed that the Al-Ce melts solidified without any cracking or porosity, and Al_11_Ce_3_ phase remained stable under the laser surface treatment. In this alloy system, there is no significant coarsening observed after a treatment at 300 °C for 24 h, indicating its superior thermal stability, which can be comparable to other excellent heat-resistant Al alloy systems like Al-Cu-Y [53], Al-Cu-Yb [54], Al-Cu-Er [55], or Al-Cu-Gd [56] alloys. Therefore, the Al-Ce alloy should also be a qualified candidate for the LPBFed Al alloy designed for the HT environment.

Although the Al-Ce binary alloy is a good candidate in novel Al alloys, there is still large space for the improvement on its mechanical performance [38]. In this sense, the alloying elements are normally required. Depending on the proportion of alloying elements, Al-Ce-based alloys can be classified as two types: (1) Al-Ce alloys modified by main-alloying elements (>1 wt.%), i.e., Cu, Mg, Si, Ni, and so on; (2) Al-Ce alloys modified by micro-alloying elements (≤1 wt.%), i.e., Sc and Zr.

## 3. Alloy Powders

In the LPBF process, the powders are the first key raw materials, which have a significant impact on the forming process of LPBFed metals. In order to achieve the ideal flowability and high density during the LPBF process, the powders should keep their spherical shape to a high degree. Powder particles with high sphericity exhibit superior flowability, which facilitates the uniform spreading of powder layers during the printing process. This, in turn, enables the formation of a more compact packing structure, thereby reducing porosity and defects in the printed parts. Additionally, the improved thermal conductivity of spherical particles ensures uniform heat transfer during laser melting, minimizing thermal stresses and residual stresses. Consequently, these factors collectively enhance the performance and reliability of the fabricated components. Currently, the predominant techniques employed for the production of spherical metal powders include plasma atomization (PA), the plasma rotating electrode process (PREP), and gas atomization (GA) [57,58,59]. Herein, the GA methods usually include vacuum induction-melting gas atomization (VIGA) and electrode induction-melting gas atomization (EIGA). Comparably, the VIGA method is the most-used technique for the production of Al alloy powders. In detail, the VIGA method uses a crucible to melt the Al alloy material, and the alloy liquid flows through the conduit at the bottom of the intermediate package to the atomization nozzle. Then, the alloy liquid is crushed by the supersonic gas impact and atomized into micron-sized droplets. Finally, the droplets are spheroidized and solidified into powders. Normally, the powders produced via VIGA show a good spherical shape, and their size distributions are usually 10–100 μm [60,61]. This range of size distribution is comparably fine. The use of finer powder particles can enhance resolution and detail precision, improve surface quality, and enhance mechanical properties. This eventually contributed to the fabrication of components with higher performance. However, the spherical powders produced via the VIGA method do feature some hollow powders and the presence of satellites in some cases. Specifically, the formation of hollow powders occurs when atomized gas becomes encapsulated within the molten droplet. Furthermore, it is anticipated that the proportion of hollow powders will increase as the particle size increases. Moreover, the satellites are formed due to the collision of metal droplets. The droplets are fragmented into smaller entities and subsequently absorbed onto the surfaces of larger particles. Notably, both hollow powders and satellites should be minimized, ensuring a successful LPBF process.

**Figure 3:**
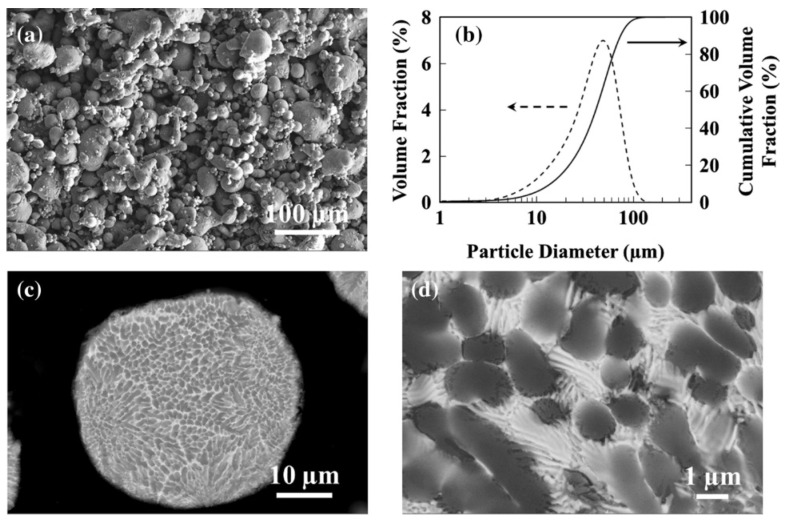
Gas-atomized Al-10Ce powders: (**a**) SEM; (**b**) powder size distribution; (**c**) BSE image in low magnification; (**d**) BSE image in high magnification [62].

For Al-Ce alloys, Zhou et al. [62] employed a specially designed laboratory-scale VIGA system to synthesize binary Al-10Ce powders. This was achieved by combining equal weights of pure aluminum and Al-20Ce master alloys. In Figure 3a, the majority of the powders exhibited a spherical morphology, although there were also instances of elongated powders and satellite particles. Also, the average powder diameter was 42.7 μm (Figure 3b). In the back-scattered electron (BSE) image (Figure 3c), the powder demonstrated a microstructure characterized by primary α-Al dendritic structures and interdendritic Al-Al_11_Ce_3_ eutectics. The sizes of both α-Al cells and Al_11_Ce_3_ compounds were smaller than those of the Al-Ce alloys experiencing arc-melting [63]. This phenomenon is likely attributable to the elevated cooling rate associated with the VIGA process.

**Figure 4:**
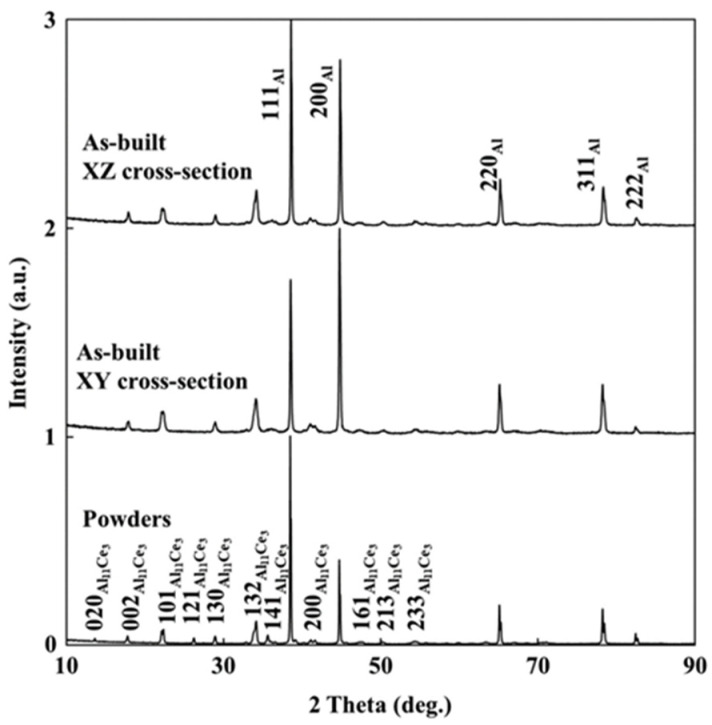
Al-10Ce XRD patterns in the gas-atomized powders: XY and XZ cross sections [62].

Figure 4 presents the X-ray diffraction (XRD) patterns of Al-10Ce powders as reported by Zhou [62]. Herein, the observed peaks were attributed to the face-centered cubic (FCC) aluminum matrix, predominantly corresponding to the (111)_Al_ and (200)_Al_ crystallographic planes. Comparably, the weaker peaks were from the Al_11_Ce_3_ with an orthorhombic crystal structure. According to the findings presented in Buschow’s research [64], the space group for Al_11_Ce_3_ is identified as *Immm*, characterized by the following lattice parameters: a = 0.4395 nm; b = 1.3025 nm; and c = 1.0092 nm. Additionally, no other phases were observed in the XRD analysis.

**Figure 5:**
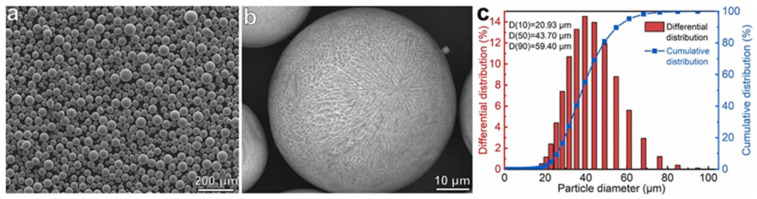
(**a**,**b**) SEM images; (**c**) size distribution of powders for the Al-9.5Ce-0.6Mg alloy [65].

In a later report, Lv et al. [65] produced the Al-9.5Ce-0.6Mg alloy powders in the near-eutectic state. The gas-atomized powders exhibited a nearly spherical morphology, devoid of irregular shapes or satellite particles. The average powder diameter was 43.70 μm for D(50) (Figure 5c). These powders were of sufficiently high quality to be conducted to the powder layered process of the LPBF treatment.

## 4. LPBF Parameters and Strategies

The LPBF process has become one of the main AM techniques for metals. The method presented offers a rapid manufacturing technique for the production of high-performance aluminum alloy components, particularly suited for applications in HT environments [66,67]. The rapid cooling rate of LPBF can dramatically enhance the microstructural refinement and facilitate the development of non-equilibrium microstructures, thereby augmenting the performance of Al alloys. However, many Al alloys are unsuitable for application in the LPBF processes due to their inadequate fluidity and high susceptibility to cracking. The cooling rate observed during the LPBF process is significantly greater than that of conventional casting methods, resulting in a comparatively elevated level of residual thermal stress. This can cause cracks, combined with the problem of fluidity. Nevertheless, the hypo-/eutectic Al-Si alloys (i.e., AlSi10Mg and AlSi12) are suitable for LPBF. The eutectic composition can reduce the cracking sensitivity by narrowing the gap between liquidus and solidus, which improves the crack resistance of Al alloys [68,69,70]. The eutectic Al-Ce system also features good flowability and a low heat crack tendency, making Al-Ce alloys candidates for LPBFed Al alloys [44,62].

For the typical LPBF process, the four primary processing parameters consist of the laser power (*P*), the scan speed (*v*), the hatch spacing (*h*), and the slice thickness (*t*). These four parameters can manipulate the local solidification behavior of alloys during the LPBF process. Furthermore, the energy density (*E*) can be constructed as the main processing coefficient, incorporating the LPBF parameters as follows [71]:

*E* = *P*/*vht*. (1)

**Figure 6:**
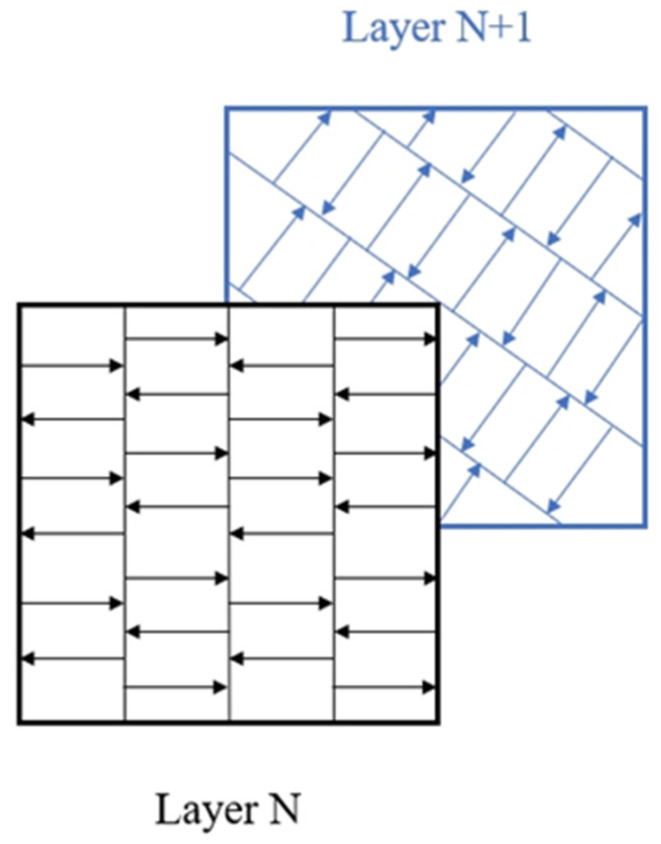
Illustration of stripe scanning.

Among these parameters, *h* and *t* are often constrained by the geometry and the LPBF facility ability. Considering the practical experiment, utilizing reduced hatch spacing is recommended to mitigate the occurrence of lack of fusion (LoF) defects. Larger hatch spacing is required to eliminate the presence of multiple remelted areas during the LPBF process. Therefore, a compromised hatch space should be achieved via an experimental test. For Al-Ce alloys, the typical parameters for hatch spacing and slice thickness are, respectively, 130 μm and 30 μm, according to previous research [62,71]. Alternative hatch spacing configurations, such as 100 μm and 190 μm, may also be employed, contingent upon the specific types of as-LPBFed Al-Ce alloys utilized. Consequently, the optimization of LPBF parameters primarily emphasizes *P* and *v*. Typically, *P* ranges from 200 W to 400 W, while the scanning speed *v* varies between 100 and 3000 mm/s. Additionally, the LPBF strategy constitutes a significant variable that can influence both the microstructure and mechanical properties of alloys produced using this method [72,73]. For example, Kudzal et al. [74] conducted an investigation into six distinct scanning patterns and their effects on the microstructure and mechanical properties of 17-4 stainless steel produced via LPBF. For as-LPBFed Al-Ce alloys, the main scan strategy is stripe scanning (Figure 6). The angle of rotation between layers is typically set at 67°, which serves to prevent the accumulation of defects aligned in a singular direction. As a result, the bonding of the layers can be in a good state [75,76].

For example, Stinehart et al. [77] applied *P* = 350 W and *v* = 1400 mm/s with the stripe scan strategy, and the relative density of the as-LPBFed Al-10Ce alloy was 99.8%. This strategy was also used in other LPBF Al-Ce-based alloys. For instance, Yang et al. [78] applied *P* = 350 W and *v* = 2000 mm/s with the stripe strategy to obtain the Al-10Ce-0.4Sc-0.2Zr alloy with a relative density of 99.92%. Although some defects were seen in certain situations, the fully or near-fully dense samples were obtained in previous research. There was no solidification cracks observed in all situations, which could be due to the good performance of Al-Ce-based alloys in terms of flowability and crack resistance, indicating the potential for producing large components with complex geometry. This is a great improvement over other heat-resistant Al alloys.

**Figure 7:**
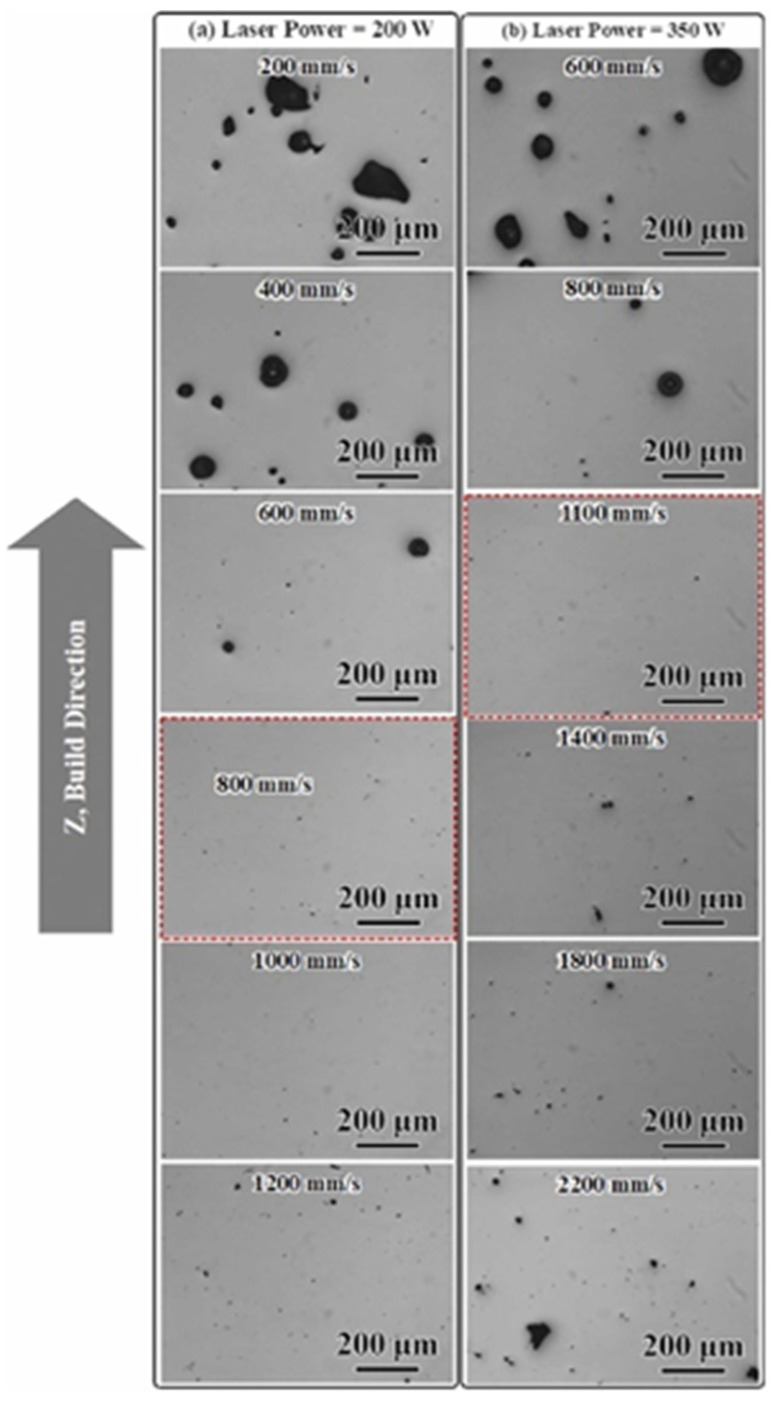
XZ plane optical images of Al-8Ce-10Mg cube samples produced with *P* = 200 W in (**a**) and 350 W in (**b**), presented as a function of scanning speed. The cubes exhibiting the highest volumetric density are delineated by red dashed line [71].

However, it is important to acknowledge that while Al-Ce-Mg alloys are considered promising candidates for the LPBF of Al-Ce alloys due to the solution strengthening effects attributed to Mg, the vaporization of Mg may adversely affect the performance characteristics of Al-Ce-Mg alloys. Hyer et al. [71] investigated the optimization of LPBF procedures for Al-8Ce-10Mg alloys. Two sets of LPBF parameters were set coordinately: *P* = 200 W, *v* = 200–1200 mm/s; and *P* = 350 W, *v* = 600–2600 mm/s. Figure 7 shows the optical images of cube samples. At the lower scan speed, the energy density (E) was higher according to Equation (1). The melting and vaporization of Mg resulted in the creation of keyholes and the occurrence of significant spherical defects. An increase in scan speed corresponded to a decrease in energy density, which facilitated the elimination of defects and the attainment of fully dense samples. However, as the scan speed was further increased, the energy density became inadequate for the fusion process, leading to the emergence of LoF defects. Ultimately, the optimized parameters identified included *v* = 800 mm/s at 200 W and *v* = 1100 mm/s at 350 W. Their relative densities were 99.3% and 98.1% for the as-LPBFed alloys, respectively.

In conclusion, the Al-Ce-based alloys without evaporable elements (i.e., Mg) exhibit great printability and high density. Al-Ce-Mg-based alloys need a specific strategy (i.e., high scan speed) to eliminate the defects, although the density is relatively lower.

## 5. Microstructure and Phase Component

### 5.1. Melt Pool

**Figure 8:**
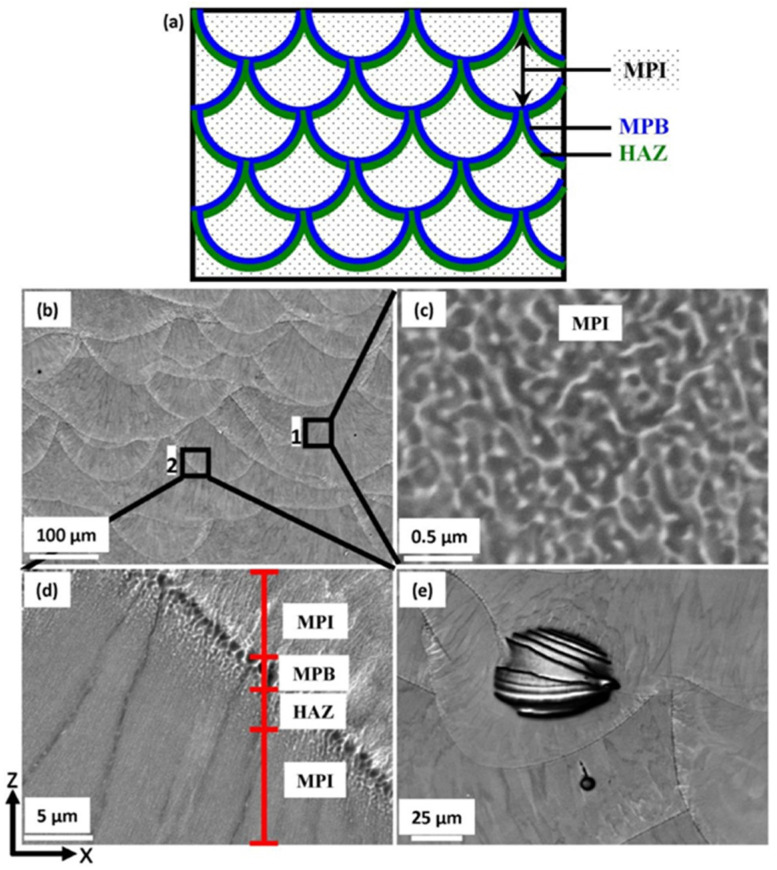
(**a**) Schematic diagram for the zones: MPI, MPB, and HAZ; (**b**–**e**) SEM images of the as-LPBFed Al-6Ce-9Cu microstructure in the XY direction: (**b**) microstructure in low magnification; (**c**) zone 1 in (**b**); (**d**) zone 2 in (**b**); (**e**) an LoF defect [80].

Throughout the construction process, the powders are subjected to elevated laser energy, resulting in their melting and the subsequent formation of a molten pool structure as the alloy solidifies. The manufacturing process of LPBF can be regarded as the stacking of melt pools by layers. Usually, the temperature gradient (*G*) and the solidification velocity (*V*) can determine the solidification conditions and thus define the structure of the melt pool. In general, the morphology of a microstructure is influenced by *G*/*V*, while the scale of the microstructure is governed by *G* × *V* [79]. In the LPBF process, the melt pool is formed in an upward direction, starting from the base and progressing to the top. Throughout the solidification phase, there is a progressive decrease in the temperature gradient, accompanied by a corresponding increase in the solidification velocity [48]. Bahl et al. [80] manufactured Al-6Ce-9Cu alloys via LPBF, and their typical microstructures along the building direction are demonstrated in Figure 8.

Figure 8a illustrates the configuration of the melt pool, which comprises the melt pool interior (MPI), the melt pool boundary (MPB), and the heat-affected zone (HAZ). The melt started solidification in the MPB and grew into the MPI. Furthermore, HAZ can be considered as a transition region between MPI and MPB, where the remelting process usually occurs. The melt pools typically exhibited a layer-by-layer configuration, as illustrated in Figure 8b. In the microstructural analysis of this alloy, a refined eutectic microstructure was identified, characterized by a dark region representing the α-Al matrix and a bright region corresponding to the intermetallic phase. Such a phase was in the shape of a partially connected network, which was different from the microstructures in MPB and HAZ. In the MPB, the grain size was significantly larger than that observed in the MPI, as illustrated in Figure 8d. This finding is consistent with the results obtained from other Al-Ce-based alloys [65,66]. In the MPB, the larger grain size could be caused by the lower solidification velocity and the in situ annealing of the previous layer, which is similar to the HAZ. This disparity suggests the presence of microstructural heterogeneity within the alloy, which may offer opportunities for enhancing the mechanical performance of Al-Ce-based alloys.

**Figure 9:**
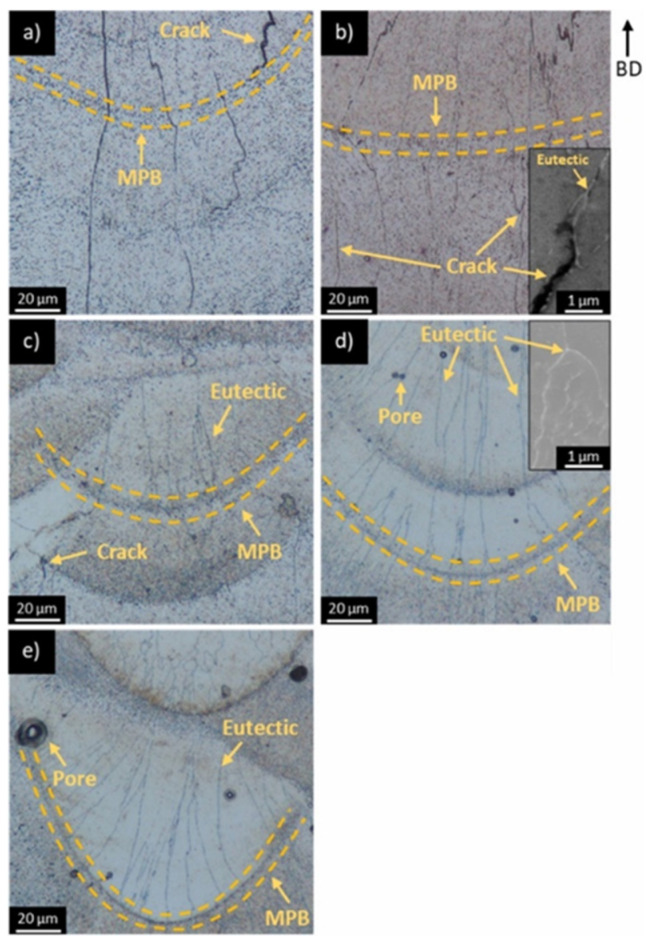
Images of melt pools for (**a**) Al6061-0Ce; (**b**) Al6061-1Ce; (**c**) Al6061-2Ce; (**d**) Al6061-3Ce; (**e**) Al6061-4Ce in the as-LPBFed state [81].

Furthermore, the concentration of Ce in Al-Ce-based alloys may influence the characteristics of the melt pools. Yakubov et al. [81] conducted an investigation into the influence of Ce concentration on the defects present in the aluminum alloy 6061 with Ce (Al6061-Ce) that underwent the LPBF process. The results are shown in Figure 9. For the Al6061 alloy, the solidification cracks were wildly distributed in MPI, MPB, and HAZ microstructures. Meanwhile, the crack directions were mostly the radial directions of the MPB, which was a typical feature of as-LPBFed Al6061 alloys. With the increasing Ce content, the intergranular eutectics suppressed the formation of solidification cracks, while the porosity increased. Herein, the porosity mainly consisted of the large LoF defects and small gas pores. Even though the building process was performed in the environment of inert gas, the Ce was primed to be oxidized to form oxides, which was supposed to be the cause of the LoF defects in the as-LPBFed Al6061-Ce alloys. During the melting process, cerium oxide interacted with the molten material, leading to the formation of AlCeO_3_. This reaction contributed to the suboptimal wettability of the melt during LPBF [82,83,84]. Additionally, the presence of gas pores is believed to have resulted from an irregular surface and the phenomenon of balling, which impeded the uniform distribution of the powder. As a result, the gas pores were formed instead of powder spreading in some regions. It should be mentioned that in this case, Ce is not a major element in the alloy, yet it shows the impact of Ce content on the quality of melt pools, which is still meaningful for the further design of LPBF Al-Ce alloys.

### 5.2. Phase Component

**Figure 10:**
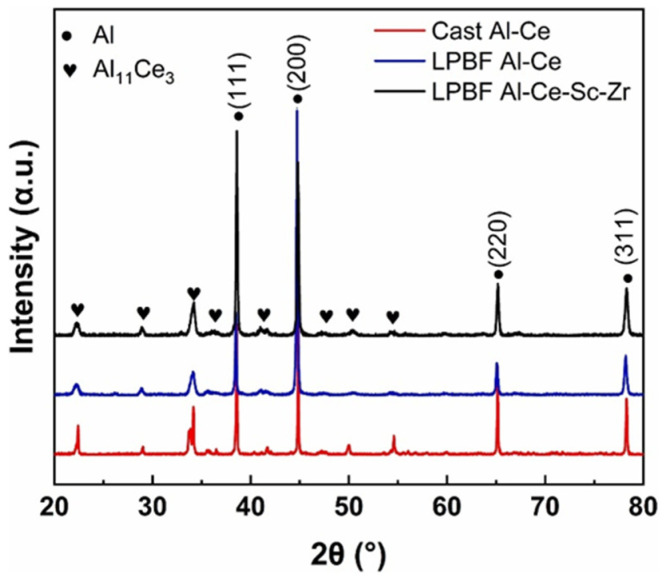
XRD patterns of the as-LPBFed Al-10Ce-0.4Sc-0.2Zr, as-LPBFed Al-10Ce, and as-cast Al-10Ce alloys [78].

For Al-Ce alloys modified by micro-alloying elements, the added elements have a negligeable impact on the main phase component. For example, the XRD patterns of as-LPBFed Al-10Ce-0.4Sc-0.2Zr, as-LPBFed Al-10Ce, and as-cast Al-10Ce alloys [78] (Figure 10) illustrate that the phases present in the three alloys are exclusively composed of an FCC Al structure and a body-centered orthorhombic Al_11_Ce_3_ structure, without other additional phases identified. This observation suggests that the incorporation of Sc and Zr does not influence the primary phase composition of the alloys produced via LPBF.

**Figure 11:**
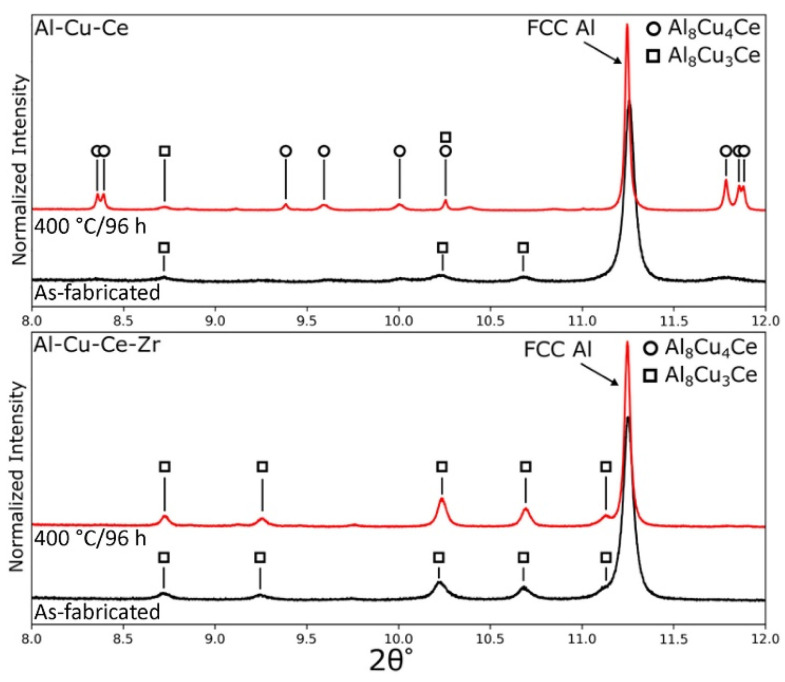
Synchrotron XRD patterns for Al-6Ce-9Cu and Al-6Ce-9Cu-1Zr alloys at 400 °C for 96 h. The crystal structure of the intermetallic phases corresponds to that of Al_24_Cu_8_Ce_3_Mn and is compositionally identified as Al_8_Cu_3_Ce. In the Al-6Ce-9Cu-1Zr alloy, these intermetallic phases undergo a transformation to Al_8_Cu_4_Ce. However, they remain stable within the Al-6Ce-9Cu-1Zr alloy at a temperature of 400 °C [80].

For Al-Ce alloys modified by main-alloying elements, the situation should be more complex, and the intermetallic phase may change depending on the element compositions. Bahl et al. [80] manufactured the Al-6Ce-9Cu alloys and the Al-6Ce-9Cu-1Zr alloys, and their synchrotron XRD patterns (Figure 11) were similar, which suggested that they had similar main phases. Herein, the FCC-Al (111) peak dominated in both alloys. This was unlike other typical Al-Ce-based alloys (i.e., the Al-10Ce alloy [62]), which owned two intense peaks of FCC-Al (111) and FCC-Al (200). The residual peaks correspond to the intermetallic compound present within the eutectic structure, which has been indexed to be Al_8_Cu_3_Ce. Furthermore, the Al_8_Cu_3_Ce is unstable in the Al-6Ce-9Cu alloy at 400 °C and can be partially converted into Al_8_Cu_4_Ce.

**Figure 12:**
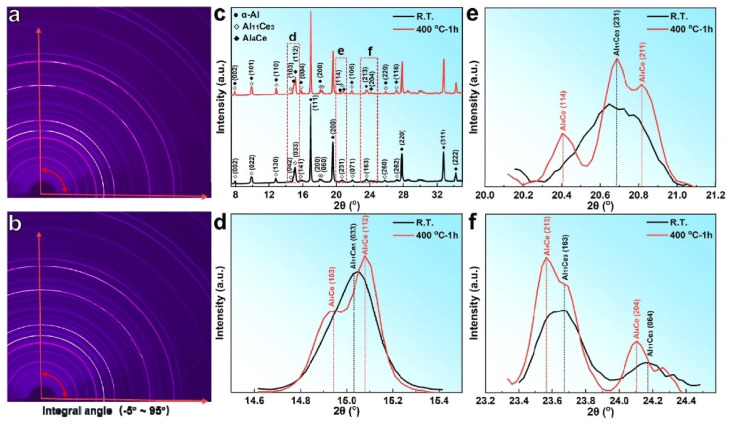
The 2D synchrotron XRD patterns for the Al-9.5Ce-0.6Mg alloy in the LPBF state (**a**) after heat treatment of 400 °C for 1 h and (**b**) before heat treatment of 400 °C for 1 h; (**c**) integrated XRD profiles; (**d**–**f**) images for the respective positions in (**c**) [85].

Recently, there have been reports emphasizing that heat treatment can have a substantial impact on the phase component of Al-Ce-based alloys. For example, Lv et al. [85] conducted ex situ SR-HRXRD tests on the as-LPBFed Al-9.5Ce-0.6Mg alloy (Figure 12a,b), and the results (Figure 12c) showed that the related RT phases were composed of α-Al and Al_11_Ce_3_ phases. However, Al_13_CeMg_6_ was not identified as a result of the insufficient Mg content present in this condition (Figure 12a,c). After 400 °C for 1 h on the as-LPBFed Al-9.5Ce-0.6Mg alloy, a phase transformation occurred, leading to the emergence of the Al_4_Ce phase (Figure 12b,c). Distinctive characteristic peaks of Al_4_Ce were observed at approximately 2θ = 15°, which included the (103) Al_4_Ce, (112) Al_4_Ce, (114) Al_4_Ce, and (204) Al_4_Ce peaks (Figure 12d–f). Using differential scanning calorimetry (DSC), the further validation of phase transformation was confirmed, and the phase transformation temperature was ~435.1 °C. The equilibrium phase diagram indicated that the heat exposure temperature of 400 °C was substantially lower than the phase transformation temperature of 1000 °C for Al_4_Ce [86,87]. This phenomenon can be attributed to the exceptionally rapid cooling rate associated with the LPBF process, leading to a solidification process that significantly diverges from equilibrium conditions. The rapid cooling rate of the LPBF technique prevents the material from reaching thermodynamic equilibrium, thereby forming microstructures that are not achievable with conventional methods, such as nanoscale precipitates [22]. Concurrently, the repeated thermal cycles introduce uneven thermal expansion and contraction, significantly elevating internal stresses [45]. Therefore, in contrast to traditional solidification techniques, the microstructure of materials produced via LPBF is characterized by a non-equilibrium state and elevated internal stresses. In the process of heat exposure, the enhancement of atomic diffusion leads to variations in local concentration, thereby creating conditions conducive to phase transformation.

### 5.3. Observation of Microstructures

Back-scattered electron (BSE) imaging is an electron imaging technique that relies on the SEM [88]. When an electron beam irradiates a sample, the incident electrons change direction when they are diffracted within the sample, and the electrons reflected from the sample are called back-scattered electrons [89]. BSE imaging is usually used to distinguish the different phases of samples, as the contrast in BSE imaging reflects the difference in atomic number [90]. Electron back-scattered diffraction (EBSD) is a technique that uses diffracted electron beams to identify the crystallographic orientation of a sample, which is wildly used to determine the grain boundary (GB), the orientation, the texture, and the strain [91,92,93]. Both BSE and EBSD images are frequently used for microstructure characterizations.

**Figure 13:**
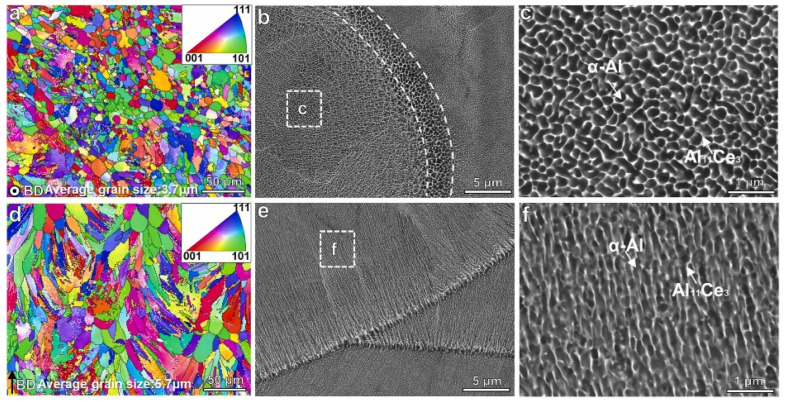
EBSD images and SEM images in BSE mode of as-LPBFed Al-9.5Ce-0.6Mg (**a**–**c**) in the SD and (**d**–**f**) in the BD: (**a**) IPF-EBSD maps; (**b**) SEM image; (**c**) SEM image in (**b**); (**d**) IPF-EBSD maps; (**e**) SEM image; (**f**) SEM image in (**e**) [65].

Usually, the microstructure predominantly comprises fine characteristics of a dark-contrast matrix alongside bright-contrast intermetallic phases within the MPI and coarser phases in the MPB. As a result, the typical “fish-scale” features are exhibited, since the difference between MPI and MPB is obvious. Perpendicular to the MPB, the directional growth of the eutectic microstructure can be attributed to the significant temperature gradient present in the LPBF process. For instance, the microstructures of as-LPBFed Al-9.5Ce-0.6Mg alloy are shown in Figure 13 with the SEM and EBSD micrographs [65]. The inverse pole figure (IPF) map of EBSD revealed that the equiaxed Al grains were randomly distributed along the scanning direction, and the mean grain size was measured to be 3.7 μm (Figure 13a). The grains displayed a columnar morphology as a result of the different temperature gradients in the building direction (Figure 13d). Once the LPBF strategy of stripe scanning was adopted, no obviously preferred orientation was detected, and the mean grain size was measured to be 5.7 μm (Figure 13d). The BSE images indicated that there was a co-continuous structure formed for the network and cell at the nanometer scale, consisting of the interconnected Al cells and Al_11_Ce_3_ intermetallics as the cell boundaries surrounding the Al matrix (Figure 13b,e). In the scanning direction, the mean diameter of the cells was about 100 nm, and the mean width for the network was about 30 nm for the MPI. The mean diameter for the cells was around 300 nm for the MPB, which refers to the dotted line area of the coarse microstructure, 2 μm in width (Figure 13b,c). In the building direction, the mean diameters for the cells representing the MPI and MPB were 150 nm and 400 nm, respectively (Figure 13e,f).

**Figure 14:**
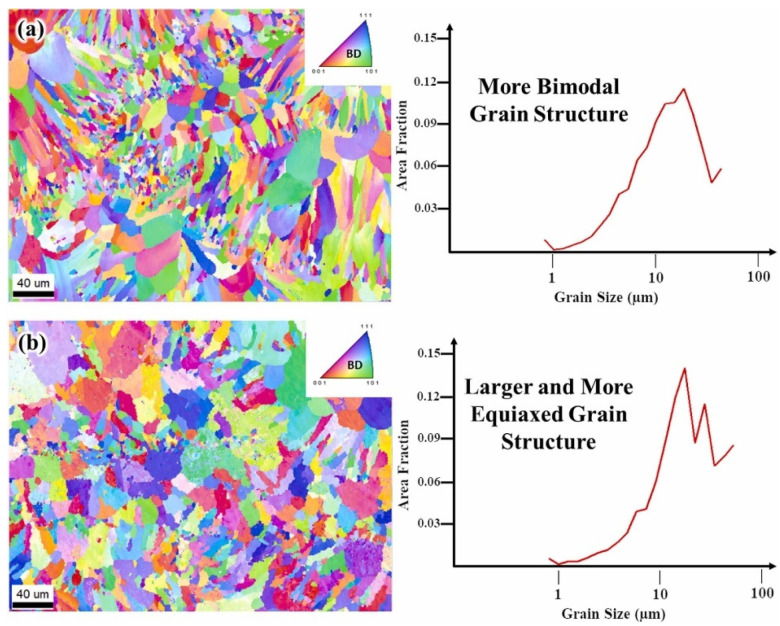
EBSD mappings for the orientation of grains and grain sizes analysis for the as-LPBFed Al-10Ce-0.4Sc-0.2Zr alloys with LPBF parameters at (**a**) *P* = 200 W and *v* = 800 mm/s and (**b**) *P* = 350 W and *v* = 1100 mm/s. The mappings illustrate the orientation of grains in relation to the building direction [71].

As well as the long columnar grains, Yang et al. [78] also identified the presence of some grains in the fine equiaxed state located at the MPB, which exhibited a bimodal grain structure characterized by both columnar and equiaxed grains. The fine equiaxed grains observed in the as-LPBFed Al-10Ce-0.4Sc-0.2Zr alloy enhanced printability by inhibiting the epitaxial growth of the columnar grains. The morphology and the size of the grains was, in fact, a function of *P* and *v*. Hyer et al. [71] conducted research into the correlation between the grain size and the printing parameters of *P* and *v*. Two combinations of parameters, with *P* = 200 W and *v* = 800 mm/s, and *P* = 350 W and *v* = 1100 mm/s, were used for EBSD analysis. The findings are presented in Figure 14. The as-LPBFed sample processed at 200 W and 800 mm/s exhibited a bimodal grain structure, characterized by fine grains in the MPB and larger grains in the MPI (Figure 14a). Conversely, the as-LPBFed sample processed at 350 W and 1100 mm/s displayed a predominance of larger equiaxed grains (Figure 14b). This phenomenon has been elucidated by Zhou et al. [94], who noted that the increased cooling rate facilitates the formation of a bimodal grain structure, whereas a reduced cooling rate results in a greater number of equiaxed grains. In contrast to the findings for the Al-9.5Ce-0.6Mg alloy reported in [65], the as-LPBFed alloy discussed in [71] exhibited a [001] texture aligned with the building direction and a near [111] orientation aligned with the scanning direction. These observations are in agreement with the results obtained for the Al-10Ce alloy [60].

High-angle annular dark field (HAADF) is a novel imaging mode of scanning transmission electron microscopy (STEM). Herein, the received electrons are mainly the incoherent scattered electrons, and the imaging contrast only reflects the change in the chemical component at different positions. Therefore, the HAADF-STEM is usually used to observe the lattice structure and atomic distribution of the sample [95,96,97]. Atom probe tomography (APT) is a high spatial method of analysis and testing for determining the atomic species, visually reconstructing their spatial positions, and showing the 3D spatial distribution of atoms of different elements in the sample [98,99]. HAADF and APT are sometimes used for further characterizations of the microstructure.

**Figure 15:**
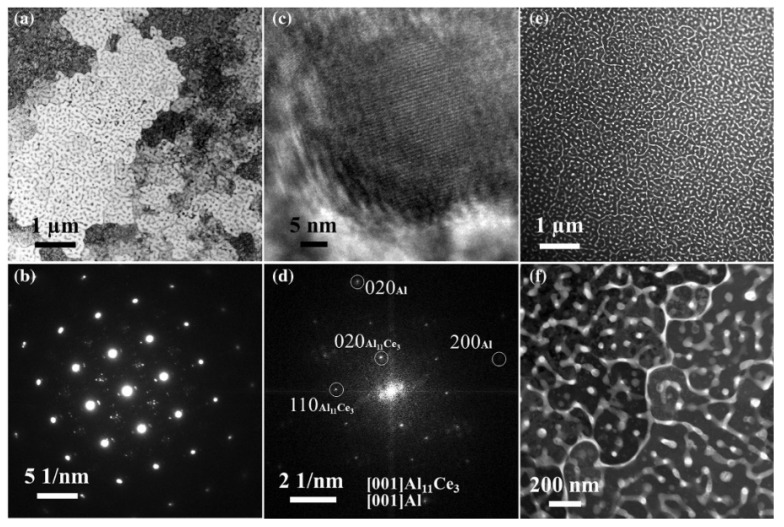
The Al-10Ce alloy in the as-LPBFed state, applying the LPBF parameter of *P* = 350 W and *v* = 1400 mm/s: (**a**) TEM image in bright field; (**b**) diffraction pattern in the specific zone; (**c**) TEM image in high resolution; (**d**) FFT from the [001]_Al_ orientation, which shows the existence of the Al_11_Ce_3_ phase; (**e**) HAADF image in low magnification; (**f**) HAADF image in high magnification [62].

Figure 15 showed the results of TEM micrographs of the as-LPBFed Al-10Ce alloy in the MPI [62]. The eutectic microstructure, characterized by the presence of two alternating phases, is depicted with greater clarity in Figure 15a. Corresponding to the XRD analysis (Figure 15b), the prominent diffraction peaks are attributed to the [001]_Al_ orientation, while the less intense diffraction peaks are associated with the Al_11_Ce_3_ phase. Fast Fourier transform (FFT) analysis shows an orientation relationship between Al and Al_11_Ce_3_: [001]_Al_‖[001]_Al11Ce3_ and (020)_Al_‖(020)_Al11Ce3_. This is consistent with the results in the Al-Ce alloys processed via arc-melting [63]. The HAADF micrograph revealed that the Al_11_Ce_3_ phase has a 10–50 nm size range, displaying a partially interconnected morphology within the Al matrix.

Moreover, the isolated skeleton particles existed in the co-continuous structure at the nano meter scale, which contributed to the reduction in the splitting Al matrix, ensuring the interconnectivity of the Al matrix [65]. The isolated particles also have potential for improving the plastic deformation performance due to their different orientations. The orientation relationship between the Al matrix and the Al_11_Ce_3_ compound can be quite different, according to different studies. For example, Yang et al. [78] showed the following orientation relationship between Al and Al_11_Ce_3_: [011]_Al_‖[731]_Al11Ce3_ and (200)_Al_‖(01-3)_Al11Ce3_. Zhang et al. [100] showed another orientation relationship: [011]_Al_‖[-113]_Al11Ce3_ and (100)_Al_‖(110)_Al11Ce3_.

**Figure 16:**
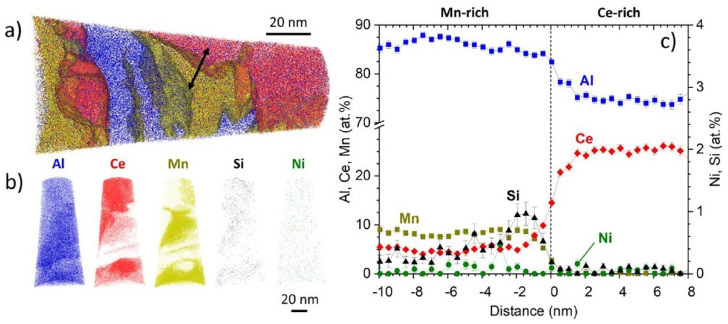
APT analysis of as-LPBFed Al10.5-Ce-3.1Ni-1.2Mn: (**a**) 3D reconstruction that illustrates individual atoms, with isoconcentration surfaces for 10 at.% Ce in red and 4 at.% Mn in yellow, respectively; (**b**) 3D atom maps that detail the spatial distribution of each atom; (**c**) a proximity histogram that elucidates the elemental distributions at the interface between a Mn-rich precipitate and a Ce-rich precipitate [101].

APT provides 3D atomic-level resolution, allowing for the precise identification and mapping of individual atoms within a material, and it can detect and analyze the low concentrations of phases, making it highly sensitive to the trace element. Therefore, APT analysis is usually carried out when the intermetallic phases are hard to determine. For example, Figure 16 presents a case of a reconstructed APT volume for the Al-10.5Ce-3.1Ni-1.2Mn alloy, wherein the interface of a Mn-rich precipitate and a Ce-rich precipitate was examined [101]. Five precipitate phases were predicted by thermodynamic calculations: Al_11_Ce_3_, Al_10_Mn_2_Ce, Al_20_Mn_2_Ce, Al_23_Ni_6_Ce_4_, and Al_3_Ni. The first four phases were well matched with the APT results. It was also mentioned that these precipitates exhibited incoherence with the Al matrix and were of a size that rendered them unsuitable for shearing. Therefore, the precise chemical compositions were not stressed in the following section on the mechanical properties.

## 6. Mechanical Properties in Room Temperature

The mechanical characteristics for alloys are typically influenced by their constituent elements and microstructural features. Alloys based on Al-Ce are primarily engineered to function as heat-resistant alloys, owing to the minimal solid solubility of Ce and its low diffusion coefficient within the Al matrix. For the conventional Al-Ce alloys, the Al_11_Ce_3_ phase exhibits relatively coarse size, leading to a poor tensile performance. Comparably, the size of the Al_11_Ce_3_ phase is in the nanometers range for the as-LPBFed Al-Ce alloys (Section 5), as they are much finer than the as-cast alloys. Table 1 shows the RT tensile properties of as-LPBFed Al-10Ce alloys compared to as-cast Al-Ce alloys [62]. For the as-LPBFed Al-10Ce alloy, the yield stress (YS) was measured at 222.1 MPa, the ultimate tensile strength (UTS) was measured at 319.3 MPa, and the elongation at fracture (EL) was measured at 10.8% (Table 1). According to Zhou et al. [62], the incremental contribution to yield strength by the Al_11_Ce_3_ phase can be assessed quantitatively through the mechanism of Orowan strengthening, which was estimated to be 193 MPa. The uniform distribution of refined intermetallic phases in the as-LPBFed alloy also contributed to the improvement of ductility. This should prevent the coarser eutectic phase from localized fracture.

Even though they exhibit enhanced properties compared to those of the Al-Ce binary alloys in the cast state (Table 1), the as-LPBFed Al-Ce binary alloys still cannot meet the requirements of commercial alloys. For instance, Wang et al. [103] exhibited that the as-LPBFed AlSi10Mg alloy owned a UTS of 478 MPa, which was higher than that of the as-LPBFed Al-10Ce alloy. The superior mechanical performance of this alloy can be ascribed to four factors: (1) the good quality of powders and the high density of samples; (2) the fine grain strengthening introduced by the high cooling rate of LPBF; (3) the Si solid-solution strengthening in the Al matrix; and (4) the fact that the Si network can be formed during the LPBF process. For the as-LPBFed Al-10Ce alloy, the effect of solid-solution strengthening is minimal, primarily due to the limited solubility of Ce in the Al matrix. Thus, the tensile performance of the alloy is limited.

For as-LPBFed Al-Ce-based alloys, the alloying method can improve the tensile performance, which can induce mechanisms that contribute to strengthening, including solid-solution strengthening and precipitation strengthening. Depending on the added elements, the mechanical properties of Al-Ce-based alloys possess the potential for enhancement across multiple dimensions. For example, the Al-Ce-Mg ternary system is one of the main alloying systems of Al-Ce alloys. The incorporation of Mg can facilitate solid-solution strengthening while not disrupting the formation of the Al_11_Ce_3_ phase during the LPBF process. Tensile mechanical tests were conducted on the as-LPBFed Al-8Ce-10Mg alloys [71]. YS = 377 MPa, UTS = 468.6 MPa, and EL = 1.8% for the samples built in 200 W, and 310 MPa (Table 2), 455.5 MPa, and 2.7% for the samples built in 350 W. The strength of the Al-8Ce-10Mg alloy was greatly improved by nearly 40%, which was similar to the Al11SiCuMn alloy [104], yet the ductility fell sharply.

Meanwhile, the Vickers hardness (HV) also decreased as the energy density increased. This phenomenon can be explained by Mg vaporization during the LPBF process under the condition of high energy density. This vaporization should be intensified due to a higher energy density, leading to a greater loss of solid-solution strengthening and a decrease in hardness and strength. Based on the microstructure observation, the Al_11_Ce_3_ phase, which is distributed at the boundary of the network, should be the main contributor to enhancing YS and UTS. The YS can be enhanced when dislocation either circumvents or penetrates a precipitate, thereby enabling the dislocations overcome the obstacle. By applying the Orowan looping mechanism, the effect of Al_11_Ce_3_ phase was estimated to be between 77 MPa and 109 MPa, while the measured YS values were 377 MPa and 310 MPa. It has been suggested that the strength of the Al-8Ce-10Mg alloy may be significantly influenced by the mechanism of solid-solution strengthening via Mg. Based on the solid-solution strengthening stress calculated by Ryen et al. [105], the contributed stress was between 168 and 171 MPa at a concentration of 6.9–7.1 wt.% Mg. The maximum stress was achieved at ~7 wt.% Mg. The contributions to YS of the impeding dislocation motion and GB strengthening were also calculated; they were less than 20 MPa and between 73 and 89 MPa, respectively. Combining these strengthening mechanisms, the theorical YS of as-the LPBFed Al-8Ce-10Mg alloy was 318–369 MPa, which complied well with the experimental result (Table 2).

In the context of micro-alloying systems involving Al-Ce alloys, the Al-Ce-Sc-Zr alloy serves as a prominent example. The incorporation of transition metal (TM) elements promotes the formation of coherent Al_3_TM precipitates characterized by an L1_2_ crystal structure [106,107,108]. Herein, among the TM elements, Sc is identified as the most advantageous for use in Al alloys. Zr typically acts as a substitute for Sc in the Al_3_Sc phase, resulting in the formation of Al_3_(Sc, Zr) and leading to a reduction in the lattice constant of Al_3_Sc, thereby enhancing its thermal stability [109]. The simultaneous addition of Sc and Zr has been demonstrated to enhance the RT strength of as-cast Al-Ce alloys effectively [110,111]. For instance, Yang et al. manufactured and studied as-LPBFed Al-10Ce-0.4Sc-0.2Zr alloy [78]. The mechanical characteristics of this alloy were found to be exceptional, with the YS of 344 MPa, UTS of 445 MPa, and EL of 10%. The Sc/Zr co-addition significantly enhanced the RT strength of the alloy. Its RT ductility can be explained by the Al_11_Ce_3_ intermetallic phase, forming the discontinuous network. The elevated strength of the material can be attributed to four distinct strengthening mechanisms: GB, solid solution, Orowan looping, and load transfer, which contributed strengths of 22 MPa, 12 MPa, 240 MPa, and 24 MPa, respectively. These findings suggest that the primary strengthening mechanism in the as-LPBFed Al-10Ce-0.4Sc-0.2Zr alloy is predominantly governed by the Orowan looping mechanism.

Furthermore, both main-alloying and micro-alloying strategies can be combined as a mixed strategy. Ekaputra et al. [112] manufactured the precipitation strengthened Al-7Ce-10Mg-0.71Zr-0.23Sc alloy. Apart from Mg as the main-alloying element and Sc/Zr as micro-alloying elements, there are also many other elements serving as alloying elements. For instance, Perrin et al. [113] studied the as-LPBFed Al-5.91Ce-8.80Cu-0.10Si-0.07Fe and Al-4.73Ce-7.78Cu-0.07Si-0.11Fe alloys, using Cu as the main-alloying element and Si and Fe as micro-alloying elements. However, the mechanical properties were not mentioned in this research and are still to be studied in further research. In conclusion, both main-alloying and micro-alloying methods can improve the performance of as-LPBFed Al-Ce alloys in terms of strength in RT, mainly through strengthening effects of the solid solution and precipitate, respectively.

## 7. Mechanical Properties in High Temperature

Al-Ce-based alloys are promising candidates for HT applications because of their good thermal stability and high eutectic temperature [114,115]. Among the mechanical properties, the creep deformation behavior in Al alloys is a crucial factor to consider in the design of components intended for extended service life. According to Michi et al. [101], it is essential to comprehend the influence of LPBF on creep behavior, particularly within the range of 200 to 450 °C. In this HT range, the as-LPBFed Al alloys are considered as alternatives to steel and Ti alloys, which have higher density and are sometimes more costly. However, the properties of resistance to creep are less promising for a few studied as-LPBFed Al alloys. For example, at the temperature of 260 °C, which is 0.57 the absolute Al melting point, the as-LPBFed Al-2.9Mg-2.1Zr alloy shows signs of creep. Consequently, the critical stress for creep exhibited a reduction of 65%, decreasing from 40 MPa to 14 MPa, following either the peak-aging treatment conducted at 400 °C or the in situ aging process that occurred during creep at 260 °C. The observed reduction in critical stress for the alloy, characterized by equiaxed fine grains, can be attributed to the phenomenon of sliding GBs, which is further facilitated by the coarsening of grains and the pinning by the particles around the GBs. In the case of the as-LPBFed Al-10Mg-0.3Si alloy subjected to a temperature of 225 °C, which is 0.53 the absolute Al melting point, with the compressive loads applied from 100 MPa to 130 MPa, the deformation contributed by creep resulted in strain rates between 1 × 10^−7^ s^−1^ and 7 × 10^−6^ s^−1^. In the process of deformation, the network of Si that was established during the LPBF process underwent coarsening and spheroidization, which was expected to contribute to the weakening of its strength. The current literature underscores two critical issues regarding the creep behavior of as-LPBFed Al alloys: (i) the presence of refined grain sizes in the alloys may promote the sliding of GBs; (ii) the necessity for strengthening phases to be highly resistant to the coarsening. Regrettably, these two characteristics are often absent from most as-LPBFed Al alloys, like the current Al-Si and Al-Si-based alloy. This suggests that alternatives to as-LPBFed Al-based alloys may present opportunities for the development of alloys with enhanced resistance to creep.

**Figure 17:**
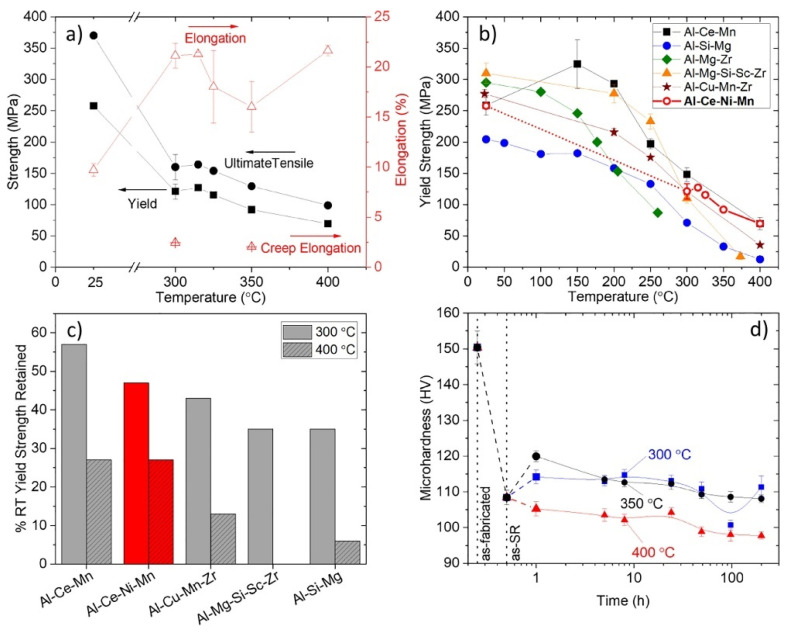
Al-10.5Ce-3.1Ni-1.2Mn alloy in the LPBF state: (**a**) YS, UTS, TEL, and CEL at different temperatures; (**b**) YS compared with other Al alloys at different temperatures; (**c**) retention of YS under an RT environment as a percentage in (**b**); (**d**) evolution of microhardness under an RT environment in the process of aging at different temperatures [101].

For instance, the as-LPBFed Al-10.5Ce-3.1Ni-1.2Mn alloy was studied recently [101]. In Figure 17a, the creep tests were performed in the as-LPBFed Al-10.5Ce-3.1Ni-1.2Mn alloy. In the conditions of creep load, the coarsened precipitates were observed to exhibit signs of increasement and gradually became quantifiable at temperatures equal to or exceeding 350 °C. In the case of the as-LPBFed Al-10.5Ce-3.1Ni-1.2Mn alloy, it is notable that the Mn element is identified as the solute with the slowest diffusion rate in this alloy. Considering a specific anticipated diffusion distance for Mn, the precipitate size measured in the condition of creep load was found to be bigger, indicating an enhancement in solute diffusion. This finding is consistent with the observations made in the rapidly solidified and consolidated Al-8.8Fe-3.7Ce alloy [116], which exhibits a microstructure analogous to that of the as-LPBFed Al-10.5Ce-3.1Ni-1.2Mn alloy.

Under the circumstance of the Al-8.8Fe-3.7Ce alloy [116], the precipitates’ improved coarsening in the condition of creep load at 17.2 MPa in 425 °C can be attributed to two primary factors: (1) the formation of vacancies surround GBs due to plastic deformation; (2) the increased solute diffusion in the area of the precipitates, which is potentially caused by diffusion within the inter-precipitate dislocations. Given that the grain size of the as-LPBFed Al-10.5Ce-3.1Ni-1.2Mn alloy, which ranges from 10 to 100 μm, is significantly larger than that of the Al-8.8Fe-3.7Ce alloy, which is around 1 μm [116], the influence of the diffusion around the GBs is potentially neglectable when it comes to the creep behavior according to the creep behavior equation in [116]:

*ε* = *C*(*λ*/*b*)^3^(*D^L^*/*b*^2^)(*σ*/*E*)^8^, (2)

where C is a material constant; b is the Burgers vector; D^L^ is the lattice self-diffusivity; σ is the stress; E the Young’s modulus; and λ is the effective dislocation barrier distance. Consequently, the coarsened precipitates during the creep process are anticipated to result primarily from solute pipe diffusion in the dislocation network established during the creep process. The pipe diffusivity exhibits an exponential increase with temperature, which aligns with the observed phenomenon of coarsened precipitate at HTs [117]. The as-LPBFed Al-10.5Ce-3.1Ni-1.2Mn alloy shows a strong property of resistance to coarsening, which underscores the potential advantages of annealing, a process traditionally regarded as unsuitable for heat treatment at temperatures exceeding the intended service temperature. In this instance, stress relief (SR) annealing was conducted at a temperature of 50 °C above the maximum testing temperature of 400 °C. This approach entails a compromise in strength, as evidenced by the reduction of 42 HV in microhardness when comparing the as-LPBFed condition to the SR condition. In this sense, the SR treatment effectively stabilizes the structure of the alloy, preventing significant alterations during subsequent aging at temperatures between 300 and 400 °C (Figure 17d). This suggests that a strategic approach to the design of heat treatment can be beneficial for enhancing the creep performance of as-LPBFed Al-Ce alloys.

Apart from the creep tests, tensile tests were also conducted on the Al-10.5Ce-3.1Ni-1.2Mn alloy at RT and HTs, as shown in Figure 17b. The YS and UTS were maximal at RT for about 260 and 370 MPa, respectively. Both values decreased with the increased temperature, while the EL displayed the opposite trend. RT ductility of around 10% was acceptable due to the high-volume of intermetallics, which are potentially fragile, in the alloy. The tensile performance in the temperature range of 100–300 °C was also tested due to the fact that some Al alloy systems, such as Al-Fe-V-Si and Al-Cr-Zr alloys, exhibited the lowest ductility in this range. For the Al-10.5Ce-3.1Ni-1.2Mn alloy in the LPBF state, there was an insignificant ductility drop in this specific temperature range. For powder metallurgy alloys, the reduction of ductility can be attributed to dynamic strain aging (DSA) from solute elements in the Al matrix, which are more active at HTs. The absence of the DSA effect within the temperature range of 100–300 °C in the as-LPBFed Al-10.5Ce-3.1Ni-1.2Mn alloy can be attributed to the majority of solute elements precipitating in the process of the cycles of temperature inherent in the LPBF process.

The microstructure of the as-LPBFed Al-10.5Ce-3.1Ni-1.2Mn alloy exhibited significant resistance to coarsening during aging at HTs, as indicated by a mere 17% increase in the mean precipitate size following 200 h of annealing, which is free of load, at 400 °C. This observation is further corroborated by the stability of the measured microhardness values post-solution treatment (Figure 17d). The remarkable coarsening resistance of the microstructure is likely attributable to the low solubilities and diffusivities of the solute elements (Ce, Ni, and Mn) within the Al matrix. For coarsening to transpire, solute atoms must migrate through the Al matrix from smaller precipitates to larger ones. Consequently, a reduced solute solubility in the Al matrix presents a thermodynamic barrier to coarsening, while diminished solute diffusivity serves as a kinetic barrier [118]. Although the diffusivity of Ni in Al at 400 °C is comparable to the self-diffusion of Al, its solubility is notably low at 0.02 at.%, thereby providing a thermodynamic impediment to coarsening [119]. In cast Al-Ni alloys, the strengthening Al_3_Ni microfibers demonstrate coarsening resistance up to approximately 400 °C [120]. In comparison, Ce exhibits an even lower solubility in Al, which is less than 0.01 at.%, and its diffusivity in Al is four orders of magnitude lower than that of Ni. Consequently, the strengthening precipitate Al_11_Ce_3_ remains resistant to coarsening for extended durations at temperatures equal to or exceeding 400 °C in cast Al-Ce alloys. Mg displays a diffusivity in Al similar to that of Ce, albeit with a solubility of 0.6 at.%. Therefore, it can be concluded that the strengthening phases present in the alloy, which are rich in Ce, Ni, and Mg, exhibit significant resistance to coarsening.

In Michi’s work [101], the analytical model can be constructed via the superposition of different strengthening mechanisms (i.e., solid-solution strengthening, GB strengthening, Orowan dislocation looping, and load transfer) in as-LPBFed Al-Ce-based alloys. For instance, in the as-SR LPBFed Al-10.5Ce-3.1Ni-1.2Mn alloy, Mn should be the only solute in matrix at an appreciable concentration. Therefore, this can determine the strengthening increment via solid-solution strengthening. The related strength elevation is as estimated by the following [105]:

Δσ_ss_ = AC_Mn_^β^, (3)

where both A = 54.8 at.%^−1^ and β = 1 are empirical constants derived from Mn in Al; and *C_Mn_* is the Mn concentration in the matrix (at.%). In the as-SR LPBFed state, the Mn concentration is 0.1 ± 0.06 at.%, as measured via APT. Then, the expected Δ*σ_ss_* is about 5 ± 3 MPa.

Moreover, the strength elevation from GBs (Δ*σ_H–P_*) can be evaluated by the classic Hall–Petch relationship [121]:

Δ*σ_H–P_* = *k_H–P_*/*d*^1/2^, (4)

where *k_H–P_* is 90 MPa; and *d* is the average grain size.

Because most grains in the alloy are non-equiaxed, the average grain size is assumed to be 10–100 μm. Based on these values, the strength elevation caused by GBs is calculated to be 19 ± 10 MPa.

Next, the strength increment provided by Orowan dislocation looping around non-shearable precipitates is given as follows [122]:

Δ*σ_Or_* = 0.4*MGb* * ln((8*<R>*/3)^1/2^/*b*)/(*π*(1 − *ν)*^1/2^*λ*), (5)

where *M* = 3.06 is the mean matrix orientation factor for Al; *b* = 0.286 nm is the magnitude of the matrix Burgers vector in Al; *ν* = 0.345 is the Poisson’s ratio for Al; *G* = 25.4 GPa is the shear modulus of pure Al; and *<R>* is the average precipitate radius. The edge-to-edge inter-precipitate distance (*λ*) is calculated from *<R>*, and the precipitate volume fraction (*ϕ*) assumes a homogenous distribution of spherical precipitates on a cubic grid:

*λ* = ((3*π*/4*ϕ*)^1/2^ − 1.64) *<R>*. (6)

Herein, *<R>* = 118 ± 29 is measured in the as-SR LPBFed state. The precipitate volume fraction is estimated to be 35%. Accordingly, the strength elevation via Orowan looping around the precipitates is 213 ± 45 MPa.

Once all the strengthening increments are summed up, the ambient-temperature YS is predicted as 264 ± 58 MPa in the as-SR LPBFed state. This value complies well with the experimental YS value (258 ± 4 MPa).

From the perspective of composite materials, the as-LPBFed Al-10.5Ce-3.1Ni-1.2Mn alloy has the high-volume fraction of precipitates, which can also be considered as a composite. Therefore, the load transfer is also expected to play a critical role in the strengthening of such material. From load transfer provided by the reinforcing phase, Hong et al. [123] estimated the effective stress on the matrix using the shear-lag model [124]:

*σ_eff_* = *σα_LT_* = *σ*(1 − (*ϕ*(*s*/2 + 1))/*ϕ(s*/2 + 1) + (1 − *ϕ*)), (7)

where *s* is the aspect ratio of the reinforcement; and *α_LT_* is the load transfer coefficient.

For the as-LPBFed Al-10.5Ce-3.1Ni-1.2Mn alloy, *ϕ* = 0.35 and *s* = 1 are used. Therefore, *α_LT_* is 0.55. The yield stress increment from load transfer (Δ*σ_LT_*) is given by the shear-lag theory of Nardone et al. [124]:

Δ*σ_LT_* = 1/2*σ_m_V_f_* (*s* + 2), (8)

where *σ_m_* is the yield stress of the matrix material without reinforcement.

However, this equation often underestimates the yield strength of material with a low-strength matrix, such as the as-SR LPBFed Al-10.5Ce-3.1Ni-1.2Mn alloy. This matrix is essentially different from pure Al. The yield strength of the matrix may be modified by including strengthening from subgrains, thermal expansion dislocations, and geometrically necessary dislocations. Assuming that the precipitate/matrix interfaces in the present material have similar strengths to the Al/SiC interface (133 MPa [125]), the maximum value for Δ*σ_LT_* is ~70 MPa. Instead, *σ_m_* = 27 MPa for the yield strength of a pure Al is used. Then, a minimum value Δ*σ_LT_* of 14 MPa is obtained. As a result, this range of Δ*σ_LT_* values (14–70 MPa) is comparable to the uncertainty in (±45 MPa), accounting for ∼80% of the YS. Indeed, the strengthening from load transfer should probably be active in the as-SR LPBFed Al-10.5Ce-3.1Ni-1.2Mn alloy in addition to solid solution, GB, and Orowan strengthening.

In comparison to other alloys, the as-LPBFed Al-10.5Ce-3.1Ni-1.2Mn alloy exhibited a relatively modest YS at RT yet demonstrated an enhanced retention of YS at HTs (Figure 17c). The lower YS at RT, when contrasted with the a-LPBFed Al-2.9Mg-2.1Zr and Al-14.1Mg-0.47Si-0.31Sc-0.17Zr alloys, can be attributed to the absence of Mg solid solution strengthening. Analytical calculations indicate that the respective Mg contents of 2.9 wt.% and 14.1 wt.% are anticipated to yield solid-solution strengthening contributions of 54 MPa and 154 MPa, respectively. Moreover, the Zr/Sc content in the latter alloys facilitates the formation of regions with refined grain structures, characterized by diameters of approximately 1 μm or less, resulting from the solidification of primary Al_3_(Sc, Zr) particles. This microstructural refinement contributes to enhanced GB strengthening in contrast to the larger grain sizes (10–100 μm) observed in the current alloy. The RT strength of the as-LPBFed Al-10.5Ce-3.1Ni-1.2Mn alloy was found to be comparable to that of the as-LPBFed Al-10Ce-8Mn and Al-8.6Cu-0.45Mn-0.90Zr alloys, which also benefit from the presence of intermetallic phase dispersions. The performance advantages of the as-LPBFed Al-10.5Ce-3.1Ni-1.2Mn alloy become evident during tensile testing at HT. In contrast to the strengthening phases presenting in other alloys, such as Mg_2_Si in Al-Mg-Si-Sc-Zr, θ-Al_2_Cu in Al-Cu-Mn-Zr, and the Si eutectic network in Al-Si-Mg, they tend to coarsen rapidly at HT and lead to diminished strengths. The coarsening resistance of the Al-10.5Ce-3.1Ni-1.2Mn alloy enables it to sustain higher yield strengths at temperatures exceeding 300 °C. Furthermore, the phenomenon of GB sliding during creep deformation appears to be inhibited in the as-LPBFed Al-10.5Ce-3.1Ni-1.2Mn alloy at temperatures up to 350 °C. This observation during creep suggests that GB sliding, which significantly contributed to the reduction in yield strength of the Al-2.9Mg-2.1Zr alloy at HT, may also be mitigated during tensile deformation of the as-LPBFed Al-10.5Ce-3.1Ni-1.2Mn alloy.

**Figure 18:**
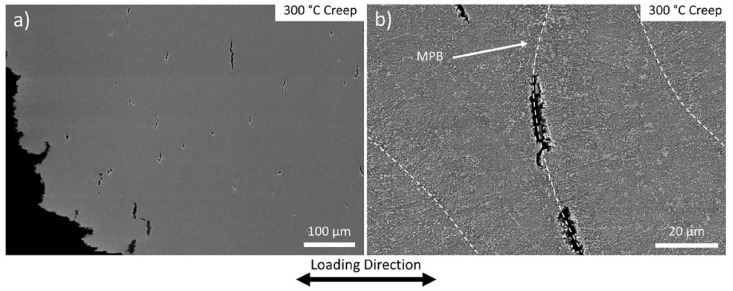
Sample that fractured during the creep test: (**a**) voids which formed away from the surface that exhibits fracture; (**b**) voids which formed around MPB [101].

Tensile tests were performed on the as-SR LPBFed Al-10.5Ce-3.1Ni-1.2Mn alloy at both RT and HT. The alloy exhibited a low creep ductility of 2% at RT, in contrast to 10% during RT tensile tests and 20% during HT tensile tests. An analysis of the creep specimens indicated that voids tended to form preferentially at the MPBs within the gauge section (Figure 18a). This observation, in conjunction with previous research on fracture behavior in additive manufactured Al-Si-Mg and Al-Cu-Ce alloys [126,127,128], suggests that the observed limited ductility during creep is primarily attributable to the preferential formation and growth of voids along the MPBs (Figure 18b).

In as-LPBFed Al-Si-Mg alloys, fractures observed during RT mechanical testing predominantly occurred along the MPBs [126,127,128]. Within the MPB and HAZ, the continuous and interconnected silicon eutectic network that developed during the printing process was disrupted and spheroidized due to the thermal effects of subsequent laser passes and material remelting. Delahaye et al. [126] suggested that the spheroidization of the silicon eutectic resulted in the formation of a locally weakened region susceptible to strain localization, dislocation accumulation, and void formation during plastic deformation, thereby diminishing the ductility of LPBF components. A similar phenomenon of strain localization was noted in the HAZ of the as-LPBFed Al-6Ce-9Cu alloy, which resulted in preferential fracture along the HAZ and a reduction in ductility during tensile testing at 300 °C. Given that the MPB structure of the as-LPBFed Al-10.5Ce-3.1Ni-1.2Mn alloy shares similarities with those of the as-LPBFed Al-Si-Mg and Al-Cu-Ce alloys, it was anticipated that a lower density of coarser precipitates relative to the surrounding material would lead to analogous strain localization. Consequently, an inter-MPB creep fracture mechanism was proposed based on this premise. During the creep deformation of the as-LPBFed Al-10.5Ce-3.1Ni-1.2Mn alloy, dislocation accumulation in the weaker MPB regions facilitated void nucleation. Under HTs and normal stress, these voids expanded through diffusional processes along MPBs, ultimately resulting in their coalescence. Once a sufficient number of voids coalesced at the MPBs, the effective stress applied to the material became sufficiently high to induce rapid ductile fracture between the coalesced MPB voids. The presence of multiple voids at MPBs exhibited varying degrees of coalescence, a theory that aligns with the observed creep fracture surfaces. The plateau-like regions corresponded to the coalesced MPB voids, with ductile fracture occurring in the intervals between these plateau regions.

Creep tests conducted with the loading axis oriented perpendicular to the building direction may provide additional insights into the creep fracture mechanism. When subjected to loading in this orientation, the nucleation and growth of voids are expected to diminish, thereby facilitating greater creep elongations. This observation aligns with the fracture surfaces identified during RT and HT tensile tests, which were executed in less than 1 h and at strain rates significantly higher than those associated with creep deformation. In the RT tests, microvoids predominantly formed along the GBs due to strain localization. However, these microvoids were unable to expand through diffusional processes at RT. Consequently, fracture occurred via microvoid coalescence driven by plastic deformation, resulting in distinct traces of the laser tracks. In contrast, the strain rates during the HT tensile tests resulted in diminished strain localization, leading to the nucleation of microvoids throughout the material. These microvoids experienced slight growth before coalescing to produce a ductile fracture, as indicated by the presence of larger microvoids. Given that the voids nucleated uniformly across the material, the microstructural features were not discernible on the fracture surface of the HT tensile test specimen. The detachment model proposed by Arzt and Rösler [129,130], along with the constant structure model developed by Sherby, Klundt, and Miller [131], has been utilized to characterize the creep behavior of dispersion-strengthened aluminum alloys with microstructures analogous to the as-LPBFed Al-10.5Ce-3.1Ni-1.2Mn alloy. This includes aluminum alloys characterized by a high-volume fraction of submicron intermetallics, such as those found in the Al-Fe-V-Si, Al-Fe-Ni, and Al-Fe-Ce systems. Both models are applicable for elucidating the creep behavior of the as-LPBFed Al-Ce-Ni-Mn alloy. It has been summarized that the combination of a high-volume fraction and the submicron size of the precipitates contributes to effective load transfer and serves as a significant barrier to dislocation motion. These factors are critical to the exceptional creep resistance exhibited by the as-LPBFed Al-10.5Ce-3.1Ni-1.2Mn alloy, contrasting with existing creep studies on Al-Mg-Si and Al-Mg-Zr alloys in the LPBF state.

The findings of this study highlight several critical factors to consider in the design of creep-resistant aluminum alloys produced via LPBF. The remarkable resistance to creep exhibited by the as-LPBFed Al-10.5Ce-3.1Ni-1.2Mn alloy indicates a high-volume fraction of coarsening-resistant and refined microstructural phases. This can be achieved during the LPBF process, serving as a promising framework for the development of future creep-resistant Al alloys in the LPBF state. Furthermore, the integration of modeling techniques and microstructural optimization facilitated by LPBF has the potential to enhance creep resistance even further. For example, among these precipitates, which one has the biggest influence on the creep performance is at all not clear. With further study on phase-specific properties via diffraction or other techniques, LPBF can be used preferentially to form an ensemble of the phases which are most creep-resistant. The alloy contains at least four distinct precipitate phases. However, it remains uncertain as to whether any specific phase demonstrates significantly greater coarsening resistance or serves as a more effective impediment to dislocation motion. Should the phase-specific properties be clarified, and suitable thermodynamic and kinetic models be established, LPBF can be employed to generate a combination of phases that exhibit optimal coarsening resistance and creep resistance selectively.

When it comes to the alloy printability in LPBF, the identification of coarsening-resistant phases that emerge during eutectic reactions of a high order represents a valuable strategy for alloy design. This identification can be facilitated through high-throughput thermodynamic calculations. Furthermore, the combination of the LPBF process with heat treatments offers a way to modify the precipitate structure. This study indicates that the spheroidization of the as-fabricated microstructure, characterized by precipitates which are less equiaxed, was achieved via the application of SR. It is likely that a trade-off exists between the stability of microstructure and load transfer, wherein more equiaxed precipitates exhibit greater resistance to coarsening, while less equiaxed precipitates serve as more effective load carriers. In the alloy microstructure examined in this study, resistance to coarsening was optimized at the expense of load transfer capabilities. Future investigations, potentially utilizing in situ neutron or XRD techniques, may further enhance creep resistance within the framework of the resistance to coarsening and load transfer trade-off. The presence of the MPB structure in as-LPBFed components is evidently a critical factor influencing creep lifetime, as demonstrated by the formation of voids along the MPBs during the creep testing of the as-LPBFed Al-Ce-Ni-Mn alloy. The LPBF processing technique can be employed to manipulate the MPB structure by altering the distribution and orientation of melt pools within the material or by controlling processing parameters to select specific phases at the MPBs that exhibit fracture resistance or inhibit void nucleation. By leveraging a microstructural template conducive to creep resistance, characterized by a high-volume fraction of coarsening-resistant and refined phases, numerous pathways for the design of creep-resistant Al alloys in the LPBF state can be explored.

**Figure 19:**
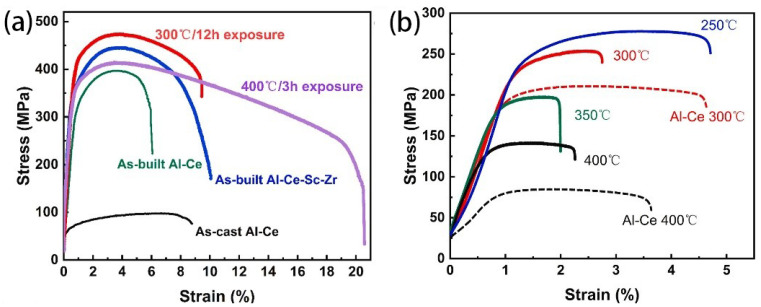
(**a**) Stress–strain curve at RT for Al-10Ce alloys and Al-10Ce-0.4Sc-0.2Zr alloys in different states; (**b**) stress–strain curve at HT for Al-10Ce-0.4Sc-0.2Zr alloys and Al-10Ce alloys [78].

In the context of micro-alloying Al-Ce alloys, Yang et al. [78] conducted tensile tests to assess the mechanical properties of the Al-10Ce-0.4Sc-0.2Zr alloy at RT and HT. In Figure 19a, the engineering stress–strain curves obtained at RT reveal a significant contrast between the as-cast Al-10Ce alloy and as-LPBFed Al-10Ce alloy. The influence of rapid solidification during the LPBF process is evident as it leads to a marked enhancement in the tensile properties of the as-LPBFed Al-10Ce alloy over its as-cast counterpart. The UTS values at RT for the as-LPBFed and as-cast Al-10Ce alloys were recorded at 397 MPa and 98 MPa, respectively. Furthermore, the incorporation of Sc and Zr elements resulted in additional improvements in tensile properties at RT. Notably, the as-LPBFed Al-10Ce-0.4Sc-0.2Zr alloy exhibited exceptional mechanical characteristics, including a UTS of 445 MPa, a YS of 344 MPa, and an EL of 10%. The ductility of the alloy at RT is also commendable, attributed to the formation of Al_11_Ce_3_ intermetallic nanoparticles that create a discontinuous network and exhibit a specific orientation relationship with the matrix.

**Figure 20:**
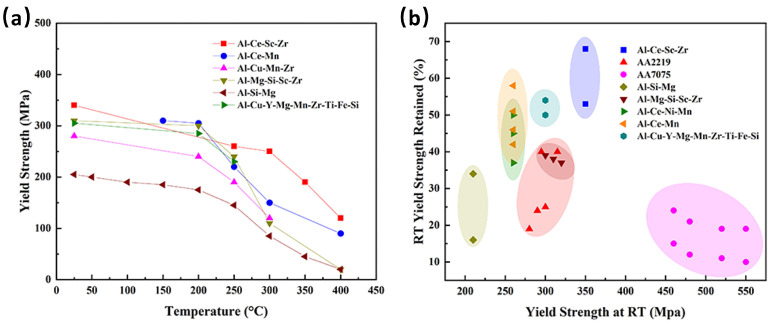
(**a**) YS at different temperatures of the as-LPBFed Al-10Ce-0.4Sc-0.2Zr alloy and other Al alloys (Al-Ce-Sc-Zr alloy [78], Al-Ce-Mn alloy [134], Al-Cu-Mn-Zr alloy [135], Al-Mg-Si-Sc-Zr alloy [76], Al-Si-Mg alloy [25], and Al-Cu-Y-Mg-Mn-Zr-Ti-Fe-Si alloy [53]); (**b**) YS retained at different temperatures of the Al-10Ce-0.4Sc-0.2Zr alloy and other Al alloys (Al-Ce-Sc-Zr alloy [78], AA2219 [136], AA7075 [137], Al-Si-Mg alloy [25], Al-Mg-Si-Sc-Zr alloy [76], Al-Ce-Ni-Mn alloy [101], Al-Ce-Mn alloy [134], and Al-Cu-Y-Mg-Mn-Zr-Ti-Fe-Si alloy [53]).

The exposure to HT facilitates the precipitation of Al_3_(Sc, Zr) from the α-Al matrix, thereby enhancing the mechanical properties of alloy. Specifically, thermal exposure at 300 °C for a duration of 12 h results in a substantial increase in the alloy’s strength with a minimal compromise to its plasticity. The UTS of the alloy reaches 474 MPa, while the YS increases to 404 MPa (Figure 19b). This performance surpasses that of the as-LPBFed Al-10Ce-0.4Sc-0.2Zr alloy at its peak-aged state, which exhibits a YS of 349 MPa [132], a UTS of 380 MPa, and a YS of 370 MPa [133]. Following a 3 h hold at 400 °C, the EL significantly improves to 20.5%, with a slight increase in YS to 356 MPa. A comparative analysis of the YS of the Al-Ce-Sc-Zr alloy with various other LPBFed Al alloys subjected to heat treatments demonstrates that the Al-10Ce-0.4Sc-0.2Zr alloy exhibits superior YS within a temperature range of 250–400 °C (Figure 20a, Al-Ce-Sc-Zr alloy [78], Al-Ce-Mn alloy [134], Al-Cu-Mn-Zr alloy [135], Al-Mg-Si-Sc-Zr alloy [76], Al-Si-Mg alloy [25], and Al-Cu-Y-Mg-Mn-Zr-Ti-Fe-Si alloy [53]). Moreover, the retention of YS at RT for the as-LPBFed Al-10Ce-0.4Sc-0.2Zr alloy is 68% at 300 °C (7% higher than that of the as-LPBFed Al-Ce alloy), 55% at 350 °C, and 38% at 400 °C (13% higher than that of the as-LPBFed Al-Ce alloy), indicating a higher retention rate over other Al alloys (Figure 20b). In consideration of the YS retention at both RT and HT, the as-LPBFed Al-10Ce-0.4Sc-0.2Zr alloy [78] demonstrates superior combined mechanical properties over most Al alloys, including AA2219 [136], AA7075 [137], Al-Si-Mg [25], Al-Mg-Si-Sc-Zr [76], Al-Ce-Ni-Mn [101], Al-Ce-Mn [134], and Al-Cu-Y-Mg-Mn-Zr-Ti-Fe-Si [53] (Figure 20b).

**Figure 21:**
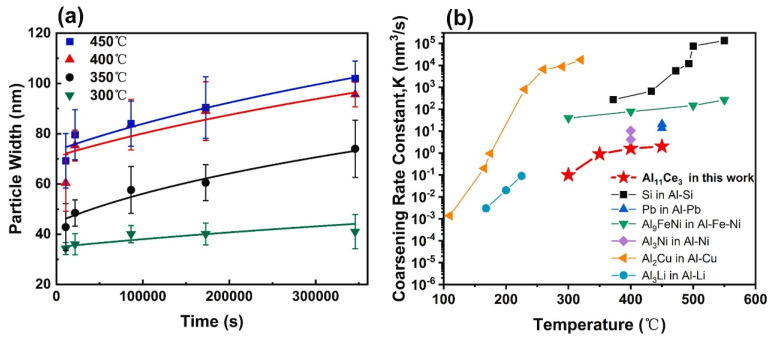
(**a**) Evolution of particle width of Al_11_Ce_3_ according to different times, varying the temperature between 300 °C and 450 °C; (**b**) coarsening rate constant (K) according to different temperatures for Al-based alloys [78].

The Al-10Ce-0.4Sc-0.2Zr alloy exhibits superior retention of mechanical properties at HT compared to other Al alloys produced via LPBF. This enhanced stability of mechanical performance is primarily attributed to the alloy’s microstructural thermal stability. In the case of the Al-10Ce-0.4Sc-0.2Zr alloy, the degree of coarsening of the eutectic Al_11_Ce_3_ phase serves as a critical determinant of the microstructural thermal stability. The kinetics of coarsening dictate the rate at which the reinforcing phase undergoes coarsening. A reduced coarsening rate of the reinforcing phase at HTs suggests that the alloy’s microstructure possesses improved thermal stability, thereby enabling it to maintain a greater proportion of its RT strength at HT. The evolution of the eutectic microstructure during prolonged heat exposure was employed to assess the size variations of the Al_11_Ce_3_ phase. The temporal changes in the average particle width of Al_11_Ce_3_ as a function of the holding time for the Al-10Ce-0.4Sc-0.2Zr alloy at temperatures ranging from 300 °C to 450 °C are illustrated in Figure 21a.

Given the intricate morphology of Al_11_Ce_3_, the particle width is approximated as the diameter *d_pw_*, which will be utilized in the subsequent calculations of coarsening kinetics. Prior research indicates that particle coarsening is typically characterized by the Lifshitz, Slyozov, and Wagner (LSW) coarsening model [138]. Additional calculation may be conducted using the following equation:

*< d_pw_*(*t*)*>*^3^ − *< d_pw_* (0)*>*^3^ = *Kt*, (9)

where *d_pw_* (0) is the preliminary average size of particles of Al_11_Ce_3_; *d_pw_* (*t*) is the average size of Al_11_Ce_3_ particles at the specific time *t*; and *K* represents the coarsening rate constant.

After applying Equation (9), the values of *K* can be obtained accordingly. The Al_11_Ce_3_ phase in the present Al-10Ce-0.4Sc-0.2Zr alloy exhibits reduced coarsening rates at HTs comparing to other prominent reinforced particles in Al alloys, like Al_9_FeNi, Al_3_Ni, Al_2_Cu, and Al_3_Li (Figure 21b). The remarkable resistance to coarsening exhibited by the Al_11_Ce_3_ phase can primarily be attributed to the minimal solubility of Ce in the Al matrix, which can be minor to 0.005% around a temperature of 640 °C, and the extremely low diffusion coefficient, which is around 5 × 10^−19^ m^2^/s at a temperature of 400 °C. Additionally, the segregation of Sc or Zr around the Al/Al_11_Ce_3_ interfaces during heat exposure may further contribute to the inhibition of coarsening in Al_11_Ce_3_ phase. A reduction in the coarsening rate of the reinforced particles during HT treatments can enhance the microstructure stability in HT environments and the mechanical performance of the Al-Ce-based alloys.

## 8. Conclusions and Perspectives

The goal of this work is to show the features of as-LPBFed Al-Ce-based alloys in terms of their microstructures and mechanical properties at both RT and HT. At this point, several aspects can be summarized:

1. Al-Ce and Al-Ce-based alloys are suitable for the development of HT alloys due to the significantly restricted solubility and diffusivity of Ce within the Al matrix. Also, Al-Ce is a eutectic system, which can improve the printability of Al-based alloys during the LPBF process.
2. The LPBF process has been demonstrated to enhance the mechanical strength of the Al-Ce alloys. For further improvement, main-alloying and micro-alloying are two promising methods that can be adopted to improve the mechanical performance of the Al-Ce alloys. Overall, Mg, Cu, Si, and Ni are the typical subjects of main-alloying strategies, and micro-alloying usually focuses on Sc and Zr.
3. Both main-alloying and micro-alloying strategies can improve strength at RT. With respect to the strengthening mechanism, the main-alloying Al-Ce alloys, i.e., the Al-Ce-Mg alloys, are strengthened by solid-solution strengthening. Micro-alloying Al-Ce alloys are strengthened via the Orowan looping mechanism in most cases. Like the typical Al-Ce-Sc-Zr alloy, the superior mechanical performance can primarily be attributed to the coherent Al_3_(Sc, Zr) particles, which can form precipitates in the matrix.
4. The mechanical performance of as-LPBFed Al-Ce-based alloys are promising in HT conditions, especially the creep resistance. The Al_11_Ce_3_ phase has excellent coarsening-resistant ability, resulting in the superior resistance of Al-Ce-based alloys. For the micro-alloying Al-Ce-based alloys, Al_3_(Sc, Zr) also help to stabilize the alloy in HT conditions. The coarsening-resistant precipitates also help to provide load transfer and to control the dislocation climb, ultimately leading to the superiority of creep resistance.

In conclusion, the advancement of as-LPBFed Al-Ce-based alloys is promising for HT applications. The fine microstructure and supersaturated solid solution resulting from the LPBF process, as well as the precipitates improving strength and creep resistance, all contribute to the excellent performance of the alloys. Furthermore, it is necessary to mention that the mechanical performance of LPBF alloys can be strongly dependent on LPBF parameters and strategies. Alloys with a promising composition need to be fabricated in optimized parameters and with the appropriate strategy.

However, the study of as-LPBFed Al-Ce alloys is still limited in many respects. Even though both main-alloying and micro-alloying methods have proven to be effective, there are still no mixed strategies that have proven to be successful because of the complexity of the various strategies and compositions. On the other hand, even though the addition of Sc/Zr is an effective method, the cost is still a problem when it comes to commercialization. The substitution of Sc/Zr can be a development direction for the commercialization of this alloy system.

## Figures and Tables

**Table 1:** RT tensile properties of some Al-Ce alloys in the cast state and the LPBF state.

Alloy	Condition	YS (MPa)	UTS (MPa)	EL (%)
Al-8Ce [102]	As-cast	40	148	19
Al-10Ce [102]	As-cast	50	152	8
Al-12Ce [102]	As-cast	57.2	161.3	13.5
Al-10Ce [62]	As-LPBFed	222.1	319.3	10.8

**Table 2:** Influence on the YS determined through the proposed strengthening mechanisms in relation to the experimental YS of Al-8Ce-10Mg alloy [71].

Strengthening	Parameters	Influence on YS (MPa)
Solid solution strengthening	6.9 wt.% Mg	168
	7.0 wt.% Mg	170
	7.1 wt.% Mg	171
Orowan looping	λ = 200 nm	109
	λ = 300 nm	77
GB strengthening	d = 200 nm	89
	d = 300 nm	73
Measured values	200 W	377
	350 W	310

## Data Availability

Not applicable.
